# Fatty acid desaturation by stearoyl-CoA desaturase-1 controls regulatory T cell differentiation and autoimmunity

**DOI:** 10.1038/s41423-023-01011-2

**Published:** 2023-04-12

**Authors:** Elien Grajchen, Melanie Loix, Paulien Baeten, Beatriz F. Côrte-Real, Ibrahim Hamad, Sam Vanherle, Mansour Haidar, Jonas Dehairs, Jelle Y. Broos, James M. Ntambi, Robert Zimmermann, Rolf Breinbauer, Piet Stinissen, Niels Hellings, Sanne G. S. Verberk, Gijs Kooij, Martin Giera, Johannes V. Swinnen, Bieke Broux, Markus Kleinewietfeld, Jerome J. A. Hendriks, Jeroen F. J. Bogie

**Affiliations:** 1 Department of Immunology and Infection, Biomedical Research Institute, Hasselt University, Diepenbeek, Belgium; 2 University MS Center Hasselt, Pelt, Belgium; 3 Neuro-Immune Connections and Repair Lab, Department of Immunology and Infection, Biomedical Research Institute, Hasselt University, Diepenbeek, Belgium; 4 VIB Laboratory of Translational Immunomodulation, VIB Center for Inflammation Research, Hasselt University, Diepenbeek, Belgium; 5 Department of Oncology, Laboratory of Lipid Metabolism and Cancer, LKI – Leuven Cancer Institute, KU Leuven - University of Leuven, Leuven, Belgium; 6 Department of Molecular Cell Biology and Immunology, Amsterdam University Medical Center, Vrije Universiteit Amsterdam, Amsterdam Neuroscience, MS Center Amsterdam, Amsterdam, The Netherlands; 7 Center for Proteomics and Metabolomics, Leiden University Medical Center, Leiden, The Netherlands; 8 Department of Biochemistry, Department of Nutritional Sciences, University of Wisconsin-Madison, Madison, USA; 9 Institute of Molecular Biosciences, University of Graz, Graz, Austria; 10 BioTechMed-Graz, Graz, Austria; 11 Institute of Organic Chemistry, Graz University of Technology, Graz, Austria; 12 Cardiovascular Research Institute Maastricht, Department of Internal Medicine, Maastricht University, Maastricht, The Netherlands

**Keywords:** Regulatory T cells, Autoimmunity, Fatty acid metabolism, Stearoyl-CoA desaturase-1, Multiple sclerosis, Autoimmunity, Lymphocyte differentiation, Lipid signalling

## Abstract

The imbalance between pathogenic and protective T cell subsets is a cardinal feature of autoimmune disorders such as multiple sclerosis (MS). Emerging evidence indicates that endogenous and dietary-induced changes in fatty acid metabolism have a major impact on both T cell fate and autoimmunity. To date, however, the molecular mechanisms that underlie the impact of fatty acid metabolism on T cell physiology and autoimmunity remain poorly understood. Here, we report that stearoyl-CoA desaturase-1 (SCD1), an enzyme essential for the desaturation of fatty acids and highly regulated by dietary factors, acts as an endogenous brake on regulatory T-cell (Treg) differentiation and augments autoimmunity in an animal model of MS in a T cell-dependent manner. Guided by RNA sequencing and lipidomics analysis, we found that the absence of *Scd1* in T cells promotes the hydrolysis of triglycerides and phosphatidylcholine through adipose triglyceride lipase (ATGL). ATGL-dependent release of docosahexaenoic acid enhanced Treg differentiation by activating the nuclear receptor peroxisome proliferator-activated receptor gamma. Our findings identify fatty acid desaturation by SCD1 as an essential determinant of Treg differentiation and autoimmunity, with potentially broad implications for the development of novel therapeutic strategies and dietary interventions for autoimmune disorders such as MS.

## Introduction

Autoimmunity reflects an imbalance between effector and regulatory mechanisms, resulting in loss of immunological self-tolerance. Multiple sclerosis (MS) is a devastating neurological disease and one of the most prevalent autoimmune disorders in the Western world. The autoimmune response in MS is characterized by an increase in autoreactive pro-inflammatory immune cell subsets, e.g., T helper (Th) 17 and Th1 cells, and a decrease in the number and function of regulatory T cells (Tregs) [1–3]. This imbalance between pathogenic and protective T cell subsets is considered to drive demyelination and neurodegeneration in the central nervous system (CNS). Hence, restoring the immune balance in favor of protective T cell subsets represents a promising strategy to halt or limit MS disease progression.

Emerging evidence indicates that fatty acids control adaptive immunity and MS disease pathology [4]. Early epidemiological studies found a strong association between excessive fat intake, obesity, and the etiology of MS [5, 6]. Notwithstanding, the direct effects of dietary fatty acids on immune cell function and CNS pathology have only recently been uncovered. Carbon chain length and degree of desaturation appear to be key determinants that dictate the immunopathological outcome of dietary fatty acids in MS, with saturated short-chain, monounsaturated long-chain, and polyunsaturated very long-chain fatty acids inducing protective immunity and reducing CNS pathology [7–10]. Several recent studies have also defined the importance of endogenous fatty acid metabolic pathways in immune cell physiology. For example, the formation of specialized proresolving lipid mediators was found to be essential for inducing a protective adaptive immune response [11, 12]. Furthermore, inhibition of de novo fatty acid synthesis and stimulation of beta-oxidation attenuate neuroinflammation and favor the differentiation of Tregs at the expense of Th17 cells [13–18]. Here, changes in the metabolism and intracellular levels of oleic (C18:1) and palmitic acid (C16:0) triggered a neuroprotective adaptive immune response [13, 18].

In this study, we defined the importance of stearoyl-CoA desaturase-1 (SCD1), an enzyme that catalyzes the rate-limiting step in the conversion of saturated fatty acids (SFAs, C16:0 and C18:0) into mono-unsaturated fatty acids (MUFAs, C16:1 and C18:1), in T- ell physiology and neuroinflammation. SCD1 belongs to the family of Δ9 fatty acid desaturases whose expression and activity are highly responsive to dietary factors and are negatively and positively regulated by polyunsaturated fatty acids (PUFAs) and Western-type diets rich in SFAs, respectively [4, 19]. We show that pharmacological inhibition of SCD1 and *Scd1* deficiency doubles peripheral Treg numbers and attenuates disease severity in experimental autoimmune encephalomyelitis (EAE), the most commonly used animal model to study MS. Guided by RNA sequencing and lipidomics analyses, we reveal that *Scd1* deficiency promotes Treg differentiation through adipose triglyceride lipase (ATGL)-dependent hydrolysis of triglycerides and phosphatidylcholine, thereby releasing nonesterified bioactive docosahexaenoic acid (DHA, C22:6), a natural ligand of the nuclear receptor peroxisome proliferator-activated receptor gamma (PPARγ). Our findings highlight the importance of SCD1 in controlling Treg differentiation and reveal its potential as a therapeutic target for MS and other autoimmune diseases.

## Materials and methods

### Mice

*Scd1*-deficient (*Scd1*^−/−^) mice and mice with the third exon of the *Scd1* gene flanked by *lox*P sites (*Scd1*^fl+/+^) have been described in previous studies [20, 21]. Both mouse strains were backcrossed at least 10 times with C57BL/6 J mice. For generation of phagocyte-specific *Scd1*^−/−^ mice, *Scd1*^fl+/+^ mice were crossbred with C57BL/6 J *LysM*^Cre^ mice, which were kindly provided by Prof. Dr. Geert van Loo (VIB-UGent Center for Inflammation Research, University of Ghent, Belgium) [22]. All mice were genotyped by PCR, as previously described [21], or according to protocols established by Jackson Laboratories (United States, Bar Harbor). In all experiments using knockout mice, wild-type (wt) littermates were used as controls. For the experiment using phagocyte-specific knockout mice, *LysM*^Cre+/−^ and *Scd1*^fl+/+^ mice were used as controls. For the adoptive transfer experiments, wt C57BL/6 J animals were purchased from Envigo (United States, Indianapolis). Mice were maintained on a 12 h light/dark cycle with free access to water and a standard chow diet.

### Experimental autoimmune encephalomyelitis (EAE)

At the age of 9–12 weeks, female mice were immunized subcutaneously with myelin oligodendrocyte glycoprotein peptide 35–55 (MOG_35–55_) emulsified in complete Freund’s adjuvant with *Mycobacterium tuberculosis* according to the manufacturer’s guidelines (EK-2110 kit; Hooke Laboratories, United States, Lawrence). Directly after immunization and after 24 h, the mice were intraperitoneally (i.p.) injected with 100 ng or 50 ng pertussis toxin (depending on lot number). Starting 5 days post-immunization, the mice with EAE were treated daily with an SCD1 inhibitor (CAY10566, 2.5 mg/kg, every 12 h by oral gavage), PPARγ inhibitor (GW9662, 2 mg/kg, every 24 h by i.p. injection), or vehicle (PBS for i.p., methylcellulose for oral gavage). Mice were weighed daily and clinically evaluated for neurological signs of the disease in a blinded fashion following a five-point standardized rating of clinical symptoms (0: no clinical symptoms; 1: tail paralysis; 2: tail paralysis and partial hind limb paralysis; 3: complete hind limb paralysis; 4: paralysis to the diaphragm; 5: death by EAE). For the adoptive transfer experiments, inguinal lymph nodes were isolated from donor mice with EAE at day 10 post-immunization. Single-cell suspensions were obtained by pushing the tissue through a 70 µm cell strainer and subsequently cultured at 7 × 10^6^ cells/ml in stimulation medium consisting of RPMI-1640 with 0.5% penicillin/streptomycin (Gibco, United States, Massachusetts), 10% FCS (Gibco), 1% nonessential amino acid (NEAA; Sigma‒Aldrich, United States, Burlington), 1% sodium pyruvate (Sigma‒Aldrich), 20 ng/ml IL23 (BioLegend; United States, San Diego), and 20 µg/ml rMOG_35–55_ (Hooke Laboratories). After three days, 10 × 10^6^ cells were intraperitoneally injected into wt or *Scd1*^−/−^ recipient mice, which were weighed and clinically evaluated for neurological signs of the disease on a daily basis and in a blinded fashion.

### Mouse T-cell cultures

CD4^+^ T-cells were isolated ex vivo from the spleens of 9- to 12-week-old wt and *Scd1*^−/−^ mice using the CD4^+^ T-Cell Isolation Kit (Miltenyi Biotec; Germany, Cologne) according to the manufacturer’s instructions. Next, live naïve (CD4^+^CD25^-^CD44^med^CD62L^+^) T cells were obtained using a FACSAria cell sorter (BD Biosciences; United States, Franklin Lakes). The purity of the isolated cells was >95%. For Treg induction, 0.75 × 10^5^ naïve T cells were cultured for one or two days with plate-bound anti-CD3ε (2 µg/ml, clone 145-2C11; BD Biosciences) in differentiation medium consisting of RPMI1640 with 1% penicillin/streptomycin, 10% FCS, 1% NEAA, 1% L-glutamine (Sigma‒Aldrich), 50 µM 2-mercaptoethanol (Gibco), anti-CD28 (2 µg/ml, clone 37.51; BD Biosciences), and rmTGFβ for Treg differentiation (10 ng/ml; R&D Systems), IL12p70 (0.02 µg/ml; R&D Systems) and αIL4 (10 µg/ml; R&D Systems) for Th1 differentiation, αIFNγ (10 µg/ml; R&D Systems) and IL4 (0.01 µg/ml; Preprotech) for Th2 differentiation, and rmTGFβ (10 ng/ml; R&D Systems) and IL6 (0.04 µg/ml; R&D Systems) for Th17 differentiation in a 96-well U-bottom plate. SCD1 inhibitor (CAY10566, 1 µM; Cayman Chemicals, United States, Michigan), ATGL inhibitor (Atglistatin, 20 µM, synthesized as described previously [23]), nonesterified DHA (C22:6, 1 µM; Cayman Chemicals), PPARα antagonist (GW6471, 25 µM; Sigma‒Aldrich), PPARβ antagonist (PT-S58, 25 µM; Sigma‒Aldrich), PPARγ antagonist (GW9662, 25 µM; Sigma‒Aldrich) or vehicle were added daily starting from the onset of cultures. DHA was dissolved in ethanol and complexed to fatty acid-free BSA (Sigma‒Aldrich) in a 4:1 molar ratio. For determination of the lipidome after ATGL inhibition, 0.75 × 10^5^ *Scd1*^−/−^ naïve T cells were cultured for two days in RPMI-1640 with 1% penicillin/streptomycin, 10% FCS, 1% NEAA, 1% L-glutamine (Sigma‒Aldrich), 50 µM 2-mercaptoethanol (Gibco), and rmIL7 (10 ng/ml; Peprotech, United Kingdom, London) in a 96-well U-bottom plate. ATGL inhibitor or vehicle was added daily starting from the onset of culture.

The suppressive capacity of wt and *Scd1*^−/−^ Tregs was assessed as described previously [24]. In brief, antigen presenting cells (APCs, CD4^-^), effector T cells (Teff, CD4^+^CD25^-^), and Tregs (CD4^+^CD25^+^) were isolated ex vivo from spleens of 11-week-old wt and *Scd1*^−/−^ animals using the CD4^+^CD25^+^ Regulatory T-Cell Isolation Kit (Miltenyi Biotec) according to the manufacturer’s instructions. The purity of the isolated cells was >95%. Increasing amounts of Tregs were cocultured alongside 0.5 × 10^5^ wt CFSE-labeled Teffs and 2 × 10^5^ irradiated wt APCs (30 Gy) in a 96-well V-bottom plate for three days. RPMI-1640 with 1% penicillin/streptomycin, 10% FCS, 1% NEAA, 1% L-glutamine, and 50 µM 2-mercaptoethanol was used as culture medium. For characterization of the T cell compartment of wt and *Scd1*^−/−^ animals with EAE, spleen and inguinal lymph nodes were isolated from the mice with EAE at day 10 post-immunization. Single-cell suspensions were obtained by pushing the tissue through a 70 µm cell strainer and cultured overnight at 5 × 10^6^ cells/ml in culture medium (RPMI-1640 with 0.5% penicillin/streptomycin, 10% FCS, 1% NEAA, and 1% sodium pyruvate).

### Human T-cell cultures

Using density gradient centrifugation, peripheral blood mononuclear cells (PBMCs) were obtained from healthy donors and age- and gender-matched relapse-remitting MS patients (untreated). CD4^+^CD25^-^ T cells were isolated using the CD4^+^ T-Cell Isolation Kit (Miltenyi Biotec) and CD25 Microbeads (Miltenyi Biotec) according to the manufacturer’s instructions. Next, recent thymic emigrated (CD4^+^CD127^+^CD31^+^, designated hereafter as naïve) T cells were obtained using a FACSAria cell sorter (BD Biosciences). The purity of the isolated cells was >95%. For Treg induction, 0.1 × 10^6^ naïve T cells were cultured for four days in differentiation medium consisting of RPMI-1640 with 0.5% penicillin/streptomycin, 10% FCS, 1% NEAA, 1% sodium pyruvate, IL2 (50 U/ml; Roche, Belgium, Brussels), rhTGFβ (5 ng/ml, Peprotech), soluble anti-CD3ε (1.25 µg/ml, clone OKT3; eBioscience, United States, San Diego), and Treg Suppression Inspector beads (Miltenyi Biotec) in a 96-well U-bottom plate. SCD1 inhibitor (CAY10566, 1 µM), ATGL inhibitor (Atglistatin, 20 µM, synthesized as described previously [23]), PPARα antagonist (GW6471, 25 µM; Sigma‒Aldrich), PPARβ antagonist (PT-S58, 25 µM; Sigma‒Aldrich), PPARγ antagonist (GW9662, 10 µM), or vehicle was added daily starting from the onset of cultures.

The suppressive capacity of Tregs was assessed as described previously [25]. In brief, effector T cells (Teff, CD4^+^CD25^-^) were stained with CellTrace Violet (CTV, Thermo Fisher Scientific, 2.5 µM) and cultured with Tregs (CD4^+^CD25^+^), which were differentiated in the presence of SCD1 inhibitor or vehicle. T cells were isolated using the CD4^+^ T-Cell Isolation Kit (Miltenyi Biotec) and CD25 Microbeads (Miltenyi Biotec) according to the manufacturer’s instructions. The purity of the isolated cells was >95%. Increasing amounts of Tregs were cocultured alongside 0.5 × 10^5^ Teff in a 96-well U-bottom plate for 5 days before being analyzed by FACS. RPMI-1640 with 0.5% penicillin/streptomycin, 10% FCS, 1% NEAA, 1% sodium pyruvate, IL2, rhTGFβ, and Treg Suppression Inspector beads was used as culture medium.

### shRNA-mediated gene silencing

Lentiviral particles were produced in HEK293T cells by cotransfecting with the packaging vector psPAX2 (8 µg, Addgene, #12260), envelope vector pMD2.G (4 µg, Addgene, #12259), and lentiviral vector (12 µg, Mission Sigma) expressing the indicated specific shRNA or a control vector expressing an unspecific shRNA using 125 mM CaCl_2_. The specific shRNA clone for ATGL was TRCN0000249774. Supernatants were collected over the course of three days and filtered through a 0.45 μm filter. Lentiviral particles were concentrated by ultracentrifugation for 2 h at 70,000 x g. Wt and *Scd1*^−/−^ naïve T cells (0.75 × 10^5^ cells/well) were stimulated for one day prior to infection. Next, T cells were transduced with lentiviral particles at a lentiviral particle medium:culture medium ratio of 1:6. One day post-infection, naïve T cells were stimulated with rmTGFβ for Treg induction. Knockdown of endogenous ATGL was confirmed using flow cytometry.

### Cell lines

Jurkat T cells were cultured in RPMI 1640 with 0.5% penicillin/streptomycin, 10% FCS, and 1% L-glutamine.

### Flow cytometry

For the staining of spleen- and lymph node-derived lymphocytes, the following antibodies were used (all purchased from BioLegend): CD45 Alexa Fluor 700 (clone 30-F11, 1:200), CD3 FITC (clone 17A2, 1:200), CD4 Pacific Blue (clone GK1.5, 1:200), CD8 BV510 (clone 53-6.7, 1:200), IFNγ PE-Cy7 (clone XMG1.2, 1:200), IL17 PE-Dazzle 594 (clone TC1-18H10.1, 1:200), IL4 PE (clone 11B11, 1:50), and FOXP3 Alexa Fluor 647 (clone MF-14, 1:200). For the isolation of mouse naïve T cells and the subsequent staining of differentiated Tregs, the following antibodies were used: CD4 PerCP-Cy5.5 (clone RM4-5, 1:100; BioLegend), CD44 APC-Cy7 (clone IM7, 1:100; BioLegend), CD25 PE-Cy7 (clone PC61, 1:200; BD Biosciences), CD62L APC (clone MEL1, 1:100; BioLegend), and FOXP3 eFluor450 (clone FJK-16s, 1:30, Invitrogen). For analysis of murine Treg suppressive capacity, the following antibodies were used: CD4 Pacific Blue (clone GK1.5, 1:200; BioLegend), CD25 PE-Cy7 (clone PC61, 1:200; BD Biosciences), and FOXP3 Alexa Fluor 647 (clone MF-14, 1:200; BioLegend). For analysis of ATGL abundance, the following antibody was used: ATGL (NB110-41536, 1:100; Novus, Belgium, Brugge). For the isolation of human naïve T cells and the subsequent staining of differentiated Tregs, the following antibodies were used: CD4 APC-eFluor780 (clone OKT4, 1:20; eBioscience), CD31 Alexa Fluor 488 (clone WM59, 1:20; BioLegend), CD127 PE (eBioRDR5, 1:20; Invitrogen), CD25 PE-Cy7 (clone M-A251, 1:40; BioLegend), and FOXP3 BV421 (clone 206D, 1:40; BioLegend). Dead cells were excluded by incubation with propidium iodide (1:250, BD Biosciences), Fixable Viability Dye eFluor506 (1:1000, Thermo Fisher Scientific), or Fixable Viability Dye Zombie NIR (1:1000, BioLegend). For analysis of surface markers, cells were stained in 1x PBS containing 2% FCS and 0.1% azide. For staining of intracellular cytokines, cells were stimulated with phorbol 12-myristate 13-acetate (20 ng/ml; Sigma‒Aldrich), calcium ionomycin (1 µg/ml; Sigma‒Aldrich), and Golgiplug (2 µg/ml; BD Biosciences) for 4 h, and the FOXP3/transcription factor staining buffer set (Invitrogen) was used according to the manufacturer’s instructions. Cells were acquired on an LSRFortessa Flow Cytometer (BD Biosciences).

### Quantitative PCR (qPCR)

Tissue or cells were lysed using QIAzol (Qiagen; The Netherlands, Roermond). RNA extraction and synthesis of complementary DNA were performed as described previously [26]. qPCR was subsequently conducted on a StepOnePlus or QuantStudio 3 detection system (Applied Biosystems; United States, Waltham). Data were analyzed using the ΔΔCt method and normalized to the most stable reference genes, as described previously [27]. Primer sequences are available on request.

### Immunofluorescence microscopy and image analysis

Cryosections were fixed in acetone for 10 min. Immunostaining and analysis of cryosections were performed as described previously [28]. The following antibodies were used: anti-CD3 (1:150; Bio-Rad, United States, Hercules), anti-F4/80 (1:100; Bio-Rad), anti-MBP (1:250; Sigma‒Aldrich), and anti-SMI312 (1:250; Biolegend) combined with Alexa Fluor 555-labeled anti-rat IgG and Alexa Fluor 488-labeled anti-mouse IgG secondary antibodies (1:400; Invitrogen, United States, Waltham). Analysis was carried out using a Nikon Eclipse 80i microscope and ImageJ software.

### Bulk RNA sequencing

Tissue or cells were lysed using QIAzol. RNA was extracted using the RNeasy mini kit (Qiagen). RNA samples were processed by the Genomics Core Leuven (Belgium). Total RNA content was analyzed with a Nanodrop 1000 spectrophotometer (Thermo Fisher Scientific), and RNA integrity was evaluated using a Bioanalyzer (Agilent). Sequencing libraries were generated using Lexogen’s QuantSeq kit and sequenced on the Illumina HiSeq4000 sequencing system, generating 50 bp single-end reads. Splice-aware alignment was performed with STAR (v2.6.1b) using the default parameters [29]. Reads mapping to multiple loci in the reference genome were discarded. Quantification of reads per gene was performed with HT-seq Count v2.7.14. For count-based differential expression analysis of a single gene, the R-based Bioconductor package DESeq2 (The R Foundation for Statistical Computing) was used. Data are available as a GEO dataset under accession numbers GSE160040 and GSE224839. Differentially expressed genes were identified based on a log_2_-fold change <−0.5 and >0.5 and a *P*-value < 0.05. The list of differentially expressed genes was used as the input for the analysis with Qiagen’s Ingenuity Pathway Analysis (IPA, Qiagen).

### Liquid chromatography‒electrospray ionization tandem mass spectrometry (LC‒ESI‒MS/MS)

Human PBMCs and CD4^+^ T cells, and mouse naïve T cell pellets were reconstituted in 700 μl of 1x PBS and mixed with 800 μl of 1 N hydrochloric acid:methanol (MeOH) 1:8 (v/v), 900 μl of chloroform, 200 μg/ml of the antioxidant 2,6-di-tert-butyl-4-methylphenol (BHT; Sigma‒Aldrich), and 3 μl of SPLASH LIPIDOMIX Mass Spec Standard (#330707; Avanti Polar Lipids, United States, Alabaster). After vortexing and centrifugation, the lower organic fraction was collected and evaporated using a Savant Speedvac spd111v (Thermo Fisher Scientific) at room temperature, and the remaining lipid pellet was stored at −20 °C under argon. The lipid pellet was reconstituted in 100% ethanol. Lipid species were analyzed by LC‒ESI‒MS/MS on a Nexera X2 UHPLC system (Shimadzu, Japan, Kioto) coupled with a hybrid triple quadrupole/linear ion trap mass spectrometer (6500+ QTRAP system; AB SCIEX, The Netherlands, Nieuwerkerk aan den IJssel). Chromatographic separation was performed on an XBridge amide column (150 mm × 4.6 mm, 3.5 μm; Waters) maintained at 35 °C using mobile phase A [1 mM ammonium acetate in H_2_O-acetonitrile 5:95 (v/v)] and mobile phase B [1 mM ammonium acetate in H_2_O-acetonitrile 50:50 (v/v)] in the following gradient: (0–6 min: 0% B → 6% B; 6–10 min: 6% B → 25% B; 10–11 min: 25% B → 98% B; 11–13 min: 98% B → 100% B; 13–19 min: 100% B; 19–24 min: 0% B) at a flow rate of 0.7 ml/min, which was increased to 1.5 ml/min from 13 min onward. Sphingomyelin (SM), cholesteryl esters (CE), ceramides (CER), dihydroceramides (DCER), hexosylceramides (HCER), and lactosylceramides (LCER) were measured in positive ion mode with precursor scans of 184.1, 369.4, 264.4, 266.4, 264.4, and 264.4, respectively. Tri-, di-, and monoacylglycerides (TGs, DGs, and MGs) were measured in positive ion mode with a neutral loss scan for one of the fatty acyl moieties. Phosphatidylcholine (PC), lysophosphatidylcholine (LPC), phosphatidylethanolamine (PE), lysophosphatidylethanolamine (LPE), phosphatidylglycerol (PG), phosphatidylinositol (PI), and phosphatidylserine (PS) were measured in negative ion mode by fatty acyl fragment ions. Lipid quantification was performed by scheduled multiple reaction monitoring (MRM), and the transitions were based on neutral losses or typical product ions as described above. The instrument parameters were as follows: curtain gas = 35 psi; collision gas = 8 a.u. (medium); IonSpray voltage = 5500 V and −4500 V; temperature = 550 °C; ion source gas 1 = 50 psi; ion source gas 2 = 60 psi; declustering potential = 60 V and −80 V; entrance potential = 10 V and −10 V; collision cell exit potential = 15 V and −15 V. Peak integration was performed with MultiQuantTM software version 3.0.3. Lipid species signals were corrected for isotopic contributions (calculated with Python Molmass 2019.1.1) and were quantified based on internal standard signals and adhered to the guidelines of the Lipidomics Standards Initiative (LSI; level 2 type quantification as defined by the LSI). Only the detectable lipid classes and fatty acyl moieties are reported in this manuscript.

### Liquid chromatography tandem mass spectrometry (LC‒MS/MS)

Protein precipitation was performed on naïve T cells by dissolving them in 500 µl of MeOH, to which 4 µl of internal standard solution consisting of [^2^H_4_]LTB_4_, [^2^H_8_]15-HETE, [^2^H_4_]PGE_2_, and [^2^H_5_]DHA (50 ng/ml) was added. Samples were placed at −20 °C for 20 min to equilibrate, after which they were spun down for 10 min (16,200 g at 4 °C). The supernatant was diluted with 2.5 ml of H_2_O, and the pH was adjusted to 3.5 using formic acid (99%). Lipids were extracted using solid-phase extraction (SPE). In short, C18 cartridges (Sep-Pak, Vac3 3 cc (200 mg)) were equilibrated with MeOH (LC‒MS grade, Merck) and H_2_O (LC‒MS grade, VWR Chemicals), after which the samples were loaded. Subsequently, cartridges were washed with H_2_O (LC‒MS grade) and *n*-hexane (Sigma‒Aldrich), followed by elution using methylformate (spectrophotometric grade, Sigma‒Aldrich). The elutes were then dried at 40 °C using a gentle stream of nitrogen and reconstituted in 100 µl of MeOH:H_2_O (40%). Next, samples were analyzed using a targeted LC‒MS/MS method as described previously [30]. Here, chromatographic separation was accomplished using a Shimadzu LC system consisting of two LC-30AD pumps, a SIL-30AC autosampler, and a CTO-20AC column oven (Shimadzu). Compounds were separated on a Kinetex C18 column (50 mm × 2.1 mm, 1.7 µm) protected with a C8 precolumn (Phenomenex, United States, Torrance). Samples were eluted at a constant flow rate of 400 µl/min with a gradient of H_2_O (eluent A) and MeOH (eluent B), both with 0.01% acetic acid [30]. Other instrument parameters were in line with the protocol of Jónasdóttir et al. [30] and scheduled MRM mode was used to detect compounds. Compounds were identified using their relative retention times together with characteristic mass transitions. These and other individually optimized parameters can be found in Supplementary Table 1. Only detectable nonesterified fatty acids and downstream metabolites are reported in this manuscript.

### Luciferase-based nuclear receptor reporter assay

For determination of the ligation of PPARα, PPARβ, and PPARγ, luciferase-based reporter assays were performed using the ONE-GloTM Luciferase Assay System kit (Promega, United States, Madison). Jurkat T cells were transfected with bacterial plasmid constructs expressing luciferase under control of the promotor region of the ligand-binding domain for PPARα, PPARβ, or PPARγ, which were kindly provided by Prof. Dr. Bart Staels (University of Lille, Inserm, France) [31, 32]. Cells were grown to 50–60% confluency in 60 mm plates, transfected with 1.8 μg of plasmid DNA including 0.2 μg pGAL4hPPARα, pGAL4hPPARβ, or pGAL4hPPARγ, 1 μg pG5-TK-GL3, and 0.6 μg of pCMV-β-galactosidase, using JetPEI (Polyplus-transfection SA, France, Illkirch-Graffenstaden) as transfection reagent. Transfected cells were treated with SCD1 inhibitor (CAY10566, 10 µM), ATGL inhibitor (Atglistatin, 20 µM), nonesterified DHA (1 µM), PPARα agonist (WY-14643, 10 µM; Sigma‒Aldrich), PPARβ agonist (GW501516, 10 µM; Sigma‒Aldrich), PPARγ agonist (Rosiglitazone, 10 µM; Millipore), or vehicle for 24 h. Following treatment, cells were lysed in lysis buffer (25 mM glycyl-glycine, 15 mM MgSO_4_, 4 mM EGTA, and 1x Triton; all from Sigma‒Aldrich). For correction for transfection efficacy, β-galactosidase activity was measured using lysate diluted 1:10 in B-gal buffer, consisting of 20% 2-nitrophenyl β-D-galactopyranoside (ONGP; Sigma‒Aldrich) and 80% Buffer Z (0.1 M Na_2_HPO_4_, 10 mM KCl, 1 mM MgSO_4_, and 3.4 µl/ml 2-mercaptoethanol; all from Sigma‒Aldrich). Luminescence and absorbance (410 nm) were measured using a FLUOstar Optima (BMG Labtech, Germany, Ortenberg).

### Statistics

Data were analyzed for statistical significance using GraphPad Prism and are reported as the mean ± SEM. Data were tested for normal distribution using the d’Agostino and Pearson omnibus normality test. When datasets were normally distributed, ANOVA (Tukey’s post hoc analysis) or a two-tailed unpaired Student’s t-test (with Welch’s correction if necessary) was used to determine statistical significance between groups. If datasets were not normally distributed, the Kruskal‒Wallis or Mann‒Whitney analysis was used. Significant differences were identified by *P*-values < 0.05 (*P < 0.05, **P < 0.01, and ***P < 0.001).

### Study approval

All animal procedures were conducted in accordance with institutional guidelines and approved by the Ethical Committee for Animal Experiments of Hasselt University (ID201521, ID201611, ID201822, ID201828, ID201912K, ID201914). All experimental protocols using PBMCs were conducted in accordance with institutional guidelines and approved by the Medical Ethical Committee of Hasselt University (UH-IMMVET-P1). Written informed consent was obtained from all participants included in this study.

## Results

### SCD1 deficiency and pharmacological inhibition reduce the severity of EAE by promoting Treg differentiation

**Fig. 1:**
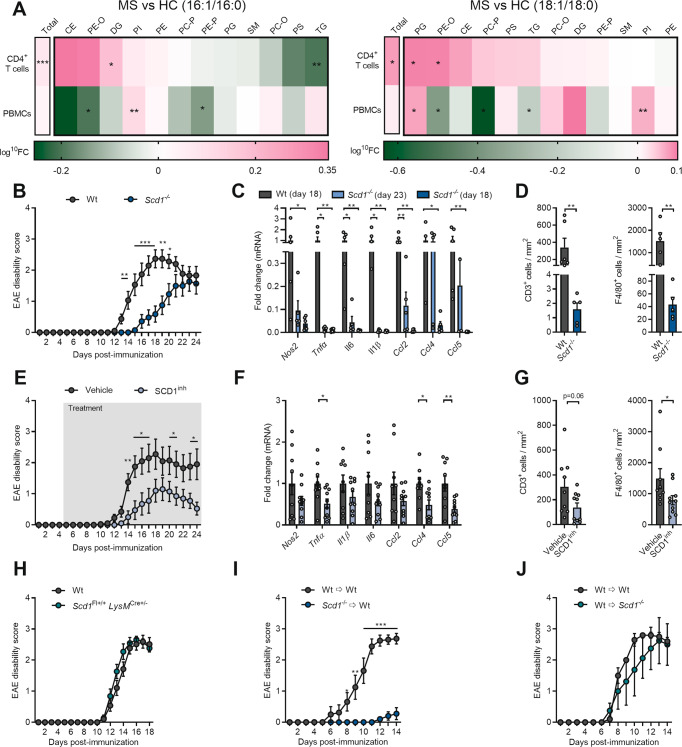
SCD1 inhibition and deficiency reduce EAE severity in a T cell-dependent manner. **A** Liquid chromatography‒electrospray ionization tandem mass spectrometry (LC‒ESI‒MS/MS) analysis was performed to define the lipidome of peripheral blood mononuclear cells (PBMCs) and CD4^+^ T cells from MS patients and age- and gender-matched healthy controls. Desaturation indices were determined by calculating the 16:1/16:0 and 18:1/18:0 ratios (*n* = 4). **B** EAE disease score of *Scd1*^−/−^ mice (*n* = 14) and wild-type littermates (wt, *n* = 15). **C** mRNA expression of *Nos2*, *Tnfα*, *Il1β*, *Il6*, *Ccl2*, *Ccl4*, and *Ccl5* in spinal cord tissue obtained from the wt (*n* = 6) and *Scd1*^−/−^ (*n* = 5) animals with EAE (18 and 23 days post-immunization, dpi). **D** Quantification of CD3 and F4/80 staining of spinal cord tissue obtained from the wt (*n* = 6) and *Scd1*^−/−^ (*n* = 5) animals with EAE at the peak of the disease (18 dpi). **E** EAE disease score of the wt mice treated with vehicle (*n* = 10) or SCD1 inhibitor (SCD1^inh^, CAY10566, 2.5 mg/kg, *n* = 10) by oral gavage twice a day with a 12 h interval. **F** mRNA expression of *Nos2*, *Tnfα*, *Il1β*, *Il6*, *Ccl2*, *Ccl4*, and *Ccl5* in spinal cord tissue obtained from the vehicle- (*n* = 9) and SCD1 inhibitor-treated (*n* = 10) animals with EAE (24 dpi). **G** Quantification of CD3 and F4/80 staining of spinal cord tissue obtained from the vehicle- (*n* = 9) and SCD1 inhibitor-treated (*n* = 10) animals with EAE (24 dpi). **H** EAE disease score of the wt (*Scd1*^Fl+/+^ and *LysM*^Cre+/-^, *n* = 19) and *Scd1*^Fl+/+^ *LysM*^Cre+/-^ (*n* = 11) mice. **I** EAE disease score of the wt recipient mice that received lymph node-derived T lymphocytes from immunized wt (Wt ➩ Wt, *n* = 8) or *Scd1*^−/−^ mice (*Scd1*^−/−^ ➩ Wt, *n* = 10). **J** EAE disease score of the wt (*n* = 5) and *Scd1*^−/−^ (*n* = 4) recipient mice that received lymph node-derived T lymphocytes from immunized wt animals. All replicates were biologically independent. All data are represented as the mean ± SEM and are pooled from 3 (**B**) or 2 (**E**, **H**, **I**) independent immunizations. *P < 0.05; **P < 0.01; ***P < 0.001; calculated with Tukey’s post hoc analysis (**B**) or a two-tailed unpaired Student’s t-test (**A**, **C**, **E**–**K**)

Endogenous and dietary-induced changes in fatty acid metabolism have a major impact on MS disease progression [5–7, 9]. Given its essential role in controlling the desaturation of fatty acids, we defined the activity of SCD1 in peripheral blood mononuclear cells (PBMCs) and CD4^+^ T cells of untreated RR-MS patients and healthy controls. LC‒ESI‒MS/MS demonstrated increased 16:1/16:0 and 18:1/18:0 desaturation indices, a proxy for SCD1 activity, within the total lipidome of CD4^+^ T cells but not PBMCs (Fig. 1A). In-depth analysis further showed that CD4^+^ T cells of MS patients displayed enhanced desaturation in diglycerides (DG), phosphatidylglycerol (PG), and phosphatidylethanolamine-O (PE-O) (Fig. 1A). Having identified enhanced SCD1 activity in CD4^+^ cells of MS patients, we next sought to define its impact on autoimmunity. To this end, the experimental autoimmune encephalomyelitis (EAE) model, an experimental MS model, was employed. By using a mouse strain deficient in *Scd1* (*Scd1*^−/−^), we found that the absence of *Scd1* attenuated EAE disease severity and incidence and significantly delayed disease onset (Fig. 1B; Supplementary Fig. 1C, D). Pathologically, *Scd1*^−/−^ mice showed a decreased inflammatory expression profile in their spinal cord compared to wt littermates, both at 18 days post-immunization and at their disease peaks (Fig. 1C). Accordingly, markedly reduced infiltration of CD3^+^ T cells and F4/80^+^ phagocytes was observed in the spinal cord of the *Scd1*^*−/−*^ mice with EAE at 18 dpi (Fig. 1D; Supplementary Fig. 1A). Consistent with a reduced neuroinflammatory burden, *Scd1*^*−/−*^ deficiency attenuated demyelination in the spinal cord at 23 days post-immunization (Supplementary Fig. 2A, B). EAE disease was not associated with alterations in axonal integrity, nor did *Scd1*^*−/−*^ deficiency affect this axonal parameter (Supplementary Fig. 2A, C). Comparable to those with *Scd1* deficiency, the animals treated with a pharmacological inhibitor of SCD1 (CAY10566) showed reduced EAE disease severity and incidence, delayed disease onset, and pathological parameters largely matched to those observed in the *Scd1*^−/−^ mice (Fig. 1E–G; Supplementary Fig. 1E, F).

Previously, we demonstrated that *Scd1* deficiency induces a less-inflammatory, repair-promoting phenotype in macrophages and microglia in the CNS [26]. To assess whether these cells are responsible for reduced EAE disease severity in *Scd1*^−/−^ mice, we induced EAE in mice with macrophage/microglia-specific *Scd1* deficiency (*Scd1*^Fl+/+^ *LysM*^Cre+/-^). Interestingly, we found no difference in EAE disease severity, incidence, and onset between the wt and *Scd1*^Fl+/+^ *LysM*^Cre+/-^ mice (Fig. 1H; Supplementary Fig. 1G, H). These findings indicate that the attenuation of EAE symptoms by *Scd1* deficiency is not mediated by macrophages and microglia. To assess whether T cells are responsible for reduced EAE severity in *Scd1*^−/−^ mice, we applied an adoptive transfer EAE model. For this purpose, lymph node-derived T lymphocytes were transferred from immunized wt or *Scd1*^−/−^ mice to wt recipient mice. Strikingly, the animals that received *Scd1*^−/−^ T cells showed almost no EAE symptoms compared to those transplanted with wt T cells (Fig. 1I; Supplementary Fig. 1I, J), mirroring the reduced demyelination in these animals (Supplementary Fig. 2A, D). No changes were observed in axonal integrity (Supplementary Fig. 2A, E). Notably, we observed no difference in EAE disease severity, incidence, and onset after transfer of wt T cells from immunized mice to wt or *Scd1*^−/−^ mice (Fig. 1J; Supplementary Fig. 1K, L). Altogether, these findings indicate that the absence of SCD1 ameliorates EAE disease severity in a T cell-dependent manner.

**Fig. 2:**
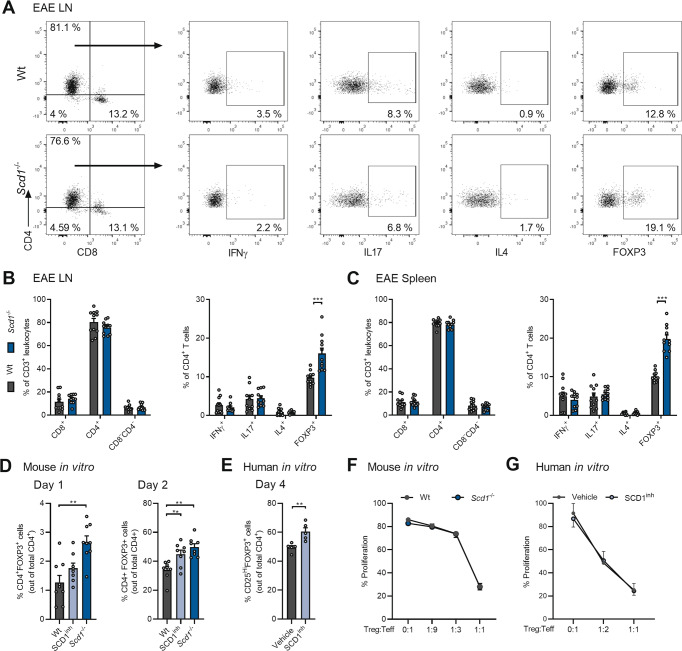
SCD1 deficiency and inhibition promote the differentiation of Tregs. **A**–**C** Frequency of CD4^+^, CD8^+^, CD4^-^CD8^-^, CD4^+^IFNγ^+^, CD4^+^IL17^+^, CD4^+^IL4^+^, and CD4^+^FOXP3^+^ cells in the lymph nodes (LN, **A**, **B**) and spleen (**C**) of wild-type (wt, *n* = 11 animals) and *Scd1*^−/−^ (*n* = 11 animals) animals with EAE 10 days post-immunization (dpi). Representative flow cytometric plots (**A**) and flow cytometric analysis (**B**, **C**) are shown. **D**, **E** Wt and *Scd1*^−/−^ mouse naïve T cells (**D**) and human naïve T cells (**E**) were differentiated under Treg polarizing conditions and treated with vehicle or SCD1 inhibitor (SCD1^inh^, CAY10566, 1 µM). For mouse T-cell cultures, the frequency of CD4^+^FOXP3^+^ cells was quantified one and two days after Treg induction (*n* = 8 samples). For human T-cell cultures, the frequency of CD25^Hi^FOXP3^+^ cells was quantified four days after induction (*n* = 5 healthy controls). The results from (**A**–**E**) are pooled from at least three independent experiments. **F**, **G** Suppressive capacity of wt and *Scd1*^*−/−*^ mouse Tregs (**F**) or human Tregs differentiated in the presence of an SCD1 inhibitor (SCD1inh, CAY10566, 1 µM) or vehicle (**G**). Increasing amounts of Tregs were cultured with CFSE- or CellTrace Violet (CTV)-labeled CD4^+^CD25^-^ effector T cells (F, *n* = 3 wt samples; **G**, *n* = 2 healthy controls). Percentage proliferation was assessed after three (**F**) or five (**G**) days. All data are represented as the mean ± SEM. *P < 0.05; **P < 0.01; ***P < 0.001, calculated with two-tailed unpaired Student’s t-test (**B**, **C**), Tukey’s post hoc analysis (**D**), or Mann‒Whitney analysis (**E**–**G**)

To define the culprit T cell subset, we characterized the peripheral T cell compartment of the wt and *Scd1*^−/−^ animals with EAE prior to disease onset. No differences were detected in the absolute number of lymph node immune cells between the wt and *Scd1*^−/−^ animals (data not shown) or in the relative abundance of CD8^+^ and CD4^+^ T cells (Fig. 2A, B). Similarly, the frequency of the T helper subsets, e.g., CD4^+^IFNγ^+^ Th1 cells, CD4^+^IL17^+^ Th17 cells, and CD4^+^IL4^+^ Th2 cells, remained unaffected in *Scd1*^−/−^ mice (Fig. 2A, B). Interestingly, we detected an increased frequency of CD4^+^FOXP3^+^ Tregs in the lymph nodes of *Scd1*^−/−^ mice (Fig. 2A, B). Similar findings were observed in the spleen of the wt and *Scd1*^−/−^ animals with EAE (Fig. 2C; Supplementary Fig. 3A). To confirm the essential role of SCD1 in T cell physiology, we next assessed the impact of SCD1 on CD4^+^ T cell subset differentiation in vitro. We observed that both genetic deficiency and pharmacological inhibition of SCD1 promoted the differentiation of Tregs from murine naïve T cells without affecting Th1, Th2, and Th17 cell differentiation (Fig. 2D; Supplementary Fig. 3B, D). A similar increase in Treg differentiation was observed using human PBMC-derived naïve T cells after exposure to the SCD1 inhibitor (Fig. 2E; Supplementary Fig. 3C). Of note, the absence or inhibition of SCD1 did not impact the suppressive capacity of mouse and human Tregs (Fig. 2F, G; Supplementary Fig. 4A, B), nor did it increase Treg proliferation (Supplementary Fig. 4C). Collectively, these findings show that SCD1 acts as a brake on the differentiation of Tregs without affecting their expansion and functional properties.

### *Scd1*-deficient naïve T cells display a transcriptional profile characteristic of Tregs

**Fig. 3:**
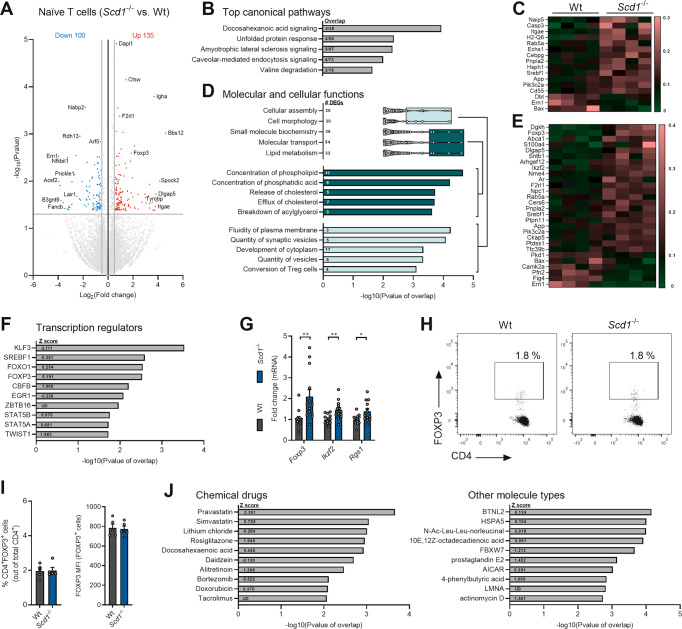
Naïve *Scd1*^−/−^ T cells display a transcriptional profile characteristic of Tregs. **A**–**F**, **J** Bulk RNA sequencing was performed using wild-type (wt) and *Scd1*^−/−^ naïve CD4^+^ T cells (CD4^+^CD25^-^CD44^med^CD62L^+^). **A** Differential gene expression analysis of the RNA sequencing data (log_2_-fold change < −0.5 and > 0.5; *P*-value < 0.05; complete list in Supplementary Table 2). **B** Top five most enriched canonical pathways in *Scd1*^−/−^ naïve T cells (complete list in Supplementary Fig. 3B). **C** Heatmap displaying the expression of genes present in the top five canonical pathways. The data were scaled by the sum of each row. **D** Molecular and cellular function categories associated with differentially expressed genes in *Scd1*^−/−^ naïve T cells. **E** Heatmap displaying the expression of genes involved in the molecular and cellular function categories. The data were scaled by the sum of each row. **F** Top 10 transcriptional regulators most associated with the transcriptome of *Scd1*^−/−^ naïve T cells. **G** mRNA expression of *Foxp3*, *Ikzf2*, and *Rgs1* in wt and *Scd1*^−/−^ naïve T cells (*n* = 11 samples) **H**, **I** The frequency of CD4^+^FOXP3^+^ cells and the respective mean fluorescence intensity of FOXP3 in wt and *Scd1*^−/−^ naïve T-cell populations were quantified (*n* = 5 samples). Representative flow cytometric plots (**H**) and flow cytometric analysis (**I**) are shown. **J** Top 10 chemical drugs, transcriptional regulators, and other molecule types most associated with the transcriptome of *Scd1*^−/−^ naïve T cells. The results are pooled from four (**A**–**F**, **I**, **J**), seven (**G**), or five (**H**) independent experiments. Data from (**G**, **I**) are represented as the mean ± SEM. *P < 0.05; **P < 0.01, calculated with two-tailed unpaired Student’s t-test (**G**, **I**)

To identify transcriptional alterations that underlie the impact of *Scd1* deficiency on T cell physiology, we performed bulk RNA sequencing. Differential gene expression analysis revealed that 235 genes distinguished wt and *Scd1*^−/−^ naïve CD4^+^ T cells, with 135 upregulated and 100 downregulated in *Scd1*^*−/−*^ naïve T cells (Fig. 3A; Supplementary Table 2). By using Ingenuity Pathway Analysis (IPA), we found that differentially expressed genes were highly associated with cancer and tumorigenic processes (Supplementary Fig. 5A), corresponding to the key role of SCD1 in cancer cell migration, metastasis, and tumor growth [33]. Canonical pathway analysis identified 34 enriched biological pathways (Supplementary Fig. 5B), including ‘DHA signaling’, ‘unfolded protein response’, and ‘amyotrophic lateral sclerosis signaling’ (Fig. 3B, C). Molecular and cellular functions that matched the transcriptional profile of naïve *Scd1*^−/−^ T cells were mapped to phospholipid, cholesterol, and acylglycerol metabolism (Fig. 3D, E). These findings confirm the essential role of SCD1 in controlling cellular lipid metabolism.

Interestingly, ‘conversion of Treg cells’ was recognized as a key cellular process in naïve *Scd1*^−/−^ T cells (Fig. 3D, E). Accordingly, based on the increased expression of *Foxp3*, *Ikzf2*, and *Rgs1*, upstream analysis demonstrated that the Treg-associated transcription factors FOXO1, FOXP3, CBFB, and STAT5 are important upstream regulators of the transcriptional signature of naïve *Scd1*^−/−^ T cells [34–37] (Fig. 3E, F). Elevated expression of Treg-associated genes such as *Foxp3*, *Ikzf2*, and *Rgs1* in naïve T cell cultures, as well as changes in the expression of other genes identified in the RNA sequencing analysis, were validated using qPCR (Fig. 3G, and Supplementary Fig. 5C). In contrast to naïve T cells, *Scd1*^−/−^ Tregs did not show altered expression of *Foxp3*, *Ikzf2*, and *Rgs1* (Supplementary Fig. 6A), mirroring their unaltered suppressive function (Fig. 2F), nor did they demonstrate changes in the expression of metabolic genes that were differentially regulated in naïve wt and *Scd1*^*−/−*^ T cells (Supplementary Fig. 6B). Likewise, bulk RNA sequencing demonstrated that wt and *Scd1*^*−/−*^ early Tregs, which were induced by exposing naïve wt and *Scd1*^*−/−*^ T cells to a Treg differentiation cocktail for a brief period of time (1 d), also did not display altered expression of these Treg-associated and metabolic genes (Supplementary Fig. 6C–E). Consistent with the latter, differentially expressed genes in early *Scd1*^*−/−*^ Tregs no longer showed an overrepresentation in those pathways that IPA identified in naïve *Scd1*^*−/−*^ T cells, including ‘conversion of Treg cells’, ‘DHA signaling’, and molecular and cellular functions related to phospholipid, cholesterol, and acylglycerol metabolism (Supplementary Fig. 6F, G). In aggregate, these findings, together with the number of FOXP3^+^ cells and FOXP3 protein abundance not differing between wt and *Scd1*^−/−^ naïve T cells (Fig. 3H, I), suggest that *Scd1* deficiency induces a transcriptional profile characteristic of Tregs in naïve T cells without affecting the transcriptome of early or committed Tregs.

Chemical drugs and other molecule types associated with the transcriptome of naïve *Scd1*^−/−^ T cells included cholesterol-reducing agents (pravastatin and simvastatin) and activators of the nuclear receptor PPARγ (rosiglitazone, docosahexaenoic acid, 10E, 12Z-octadecadienoic acid, prostaglandin E2, and 4-phenylbutyric acid) (Fig. 3J). The positive association between these lipid-modifying chemical drugs and fatty acid-containing lipid species confirms the pathway and functional enrichment analysis and further underlines the role of SCD1 in cellular lipid metabolism. In conclusion, pathway and gene set enrichment analyses indicate that *Scd1* deficiency in naïve T cells affects DHA and PPARγ signaling, alters phospholipid and triglyceride metabolism, and induces a transcriptional profile characteristic of Tregs.

### *Scd1* deficiency results in PUFA-depleted lysolecithin and triglycerides

**Fig. 4:**
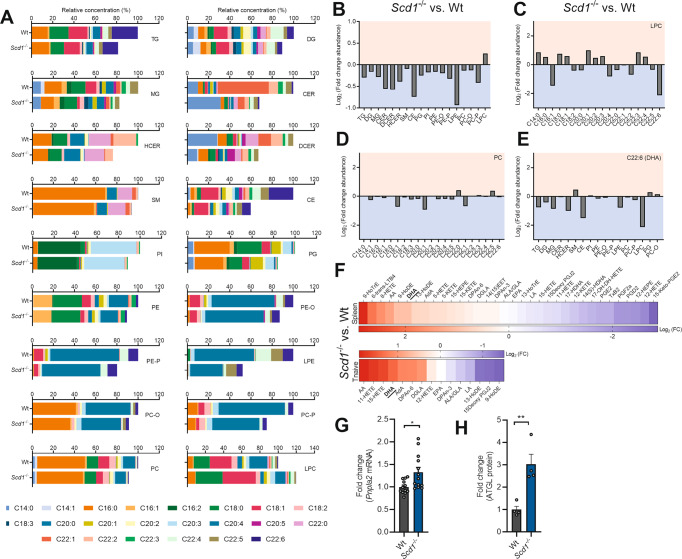
*Scd1* deficiency results in DHA-depleted lysolecithin and triglycerides. **A**–**E** Liquid chromatography‒electrospray ionization tandem mass spectrometry (LC‒ESI‒MS/MS) analysis was performed to define the lipidome of wild-type (wt) and *Scd1*^−/−^ naïve T cells (*n* = 2 samples). **A** Fatty acid composition of all lipid classes. **B**–**E** Log_2_ fold change abundance of all lipid classes (**B**), fatty acyl moieties within lysophosphatidylcholine (LPC, **C**) and phosphatidylcholine (PC, **D**), and docosahexaenoic acid (DHA) within all lipid classes (**E**) is shown (*Scd1*^−/−^ vs. wt). Only detectable fatty acyl moieties and nonesterified fatty acids and downstream metabolites are reported. **F** LC‒MS/MS analysis was performed to determine the abundance of nonesterified fatty acids and downstream metabolites in wt and *Scd1*^−/−^ naïve T cells and spleen tissue (*n* = 3 samples). Log_2_ fold change abundance of all fatty acids and downstream metabolites is shown (*Scd1*^−/−^ vs. wt). **G** mRNA expression of *Pnpla2* in wt and *Scd1*^−/−^ naïve T cells (*n* = 11 samples). **H** ATGL protein levels in wt and *Scd1*^−/−^ naïve T cells (*n* = 4 samples). The results are pooled from two (**A**–**E**, **H**), three (**F**), or seven (**G**) independent experiments. Data are represented as the mean (**A**–**F**) or as the mean ± SEM (**G**). *P < 0.05; **P < 0.01, calculated with two-tailed unpaired Student’s t-test (**G**, **H**)

Given the overrepresentation of differentially expressed genes in pathways related to lipid metabolism, liquid chromatography-electrospray ionization tandem mass spectrometry (LC‒ESI‒MS/MS) analysis was performed to define the fatty acid lipidome of wt and *Scd1*^−/−^ naïve T cells. Consistent with reduced SCD1 activity, naïve *Scd1*^−/−^ T cells demonstrated decreased 16*:*1/16:0 and C18:1/C18:0 desaturation indices, particularly within phospholipid classes (Supplementary Fig. 7A, B). Of interest, naïve *Scd1*^−/−^ T cells showed decreased levels of all major lipid classes except for lysophosphatidylcholine (LPC), a hydrolyzed derivative of phosphatidylcholine (PC; Fig. 4A, B; detailed information in Supplementary Fig. 7C–R), which argues for elevated hydrolysis of PC in naïve *Scd1*^−/−^ T cells. The overall decrease in cellular lipids in naïve *Scd1*^−/−^ T cells did not depend on reduced cell size or granularity (Supplementary Fig. 7S).

In-depth analysis further demonstrated that LPC in naïve *Scd1*^−/−^ T cells was mainly depleted of the ω3 PUFA DHA (C22:6), aside from the expected decrease in palmitoleic acid (C16:1) (Fig. 4C). This decrease in DHA in LPC was not apparent in PC (Fig. 4D). Interestingly, mono-, di-, and triacylglycerides (MGs, DGs, and TGs) also showed a marked decrease in DHA (Fig. 4E), mirroring the enrichment of genes involved in the catabolism of acylglycerols in *Scd1*^−/−^ naïve T cells (Fig. 3D). These findings point toward enhanced activity of a lipase that promotes the hydrolysis of PC and acylglycerols and favors the release of DHA in *Scd1*^−/−^ naïve T cells. In support of the latter, LC‒MS/MS analysis revealed an increased abundance of nonesterified DHA, alongside other PUFAs, in spleen tissue and naïve T cells from *Scd1*^−/−^ animals (Fig. 4F). To identify the lipase involved, we re-evaluated differentially expressed genes obtained from our transcriptomic analysis. We found that the gene expression of *Pnpla2*, which encodes the enzyme adipose triglyceride lipase (ATGL), was elevated in naïve *Scd1*^−/−^ T cells (Fig. 3C, E). Enhanced expression of ATGL in *Scd1*^−/−^ naïve T cells was validated at the gene and protein levels (Fig. 4G, H). ATGL is a calcium-independent cellular phospholipase that hydrolyses TGs and phospholipids rich in DHA and arachidonic acid (AA) [38]. With respect to the latter, *Scd1*^*−/−*^ T cells also showed a reduced abundance of AA in LPC and elevated levels of nonesterified AA (Fig. 4C, F). Collectively, these data suggest that *Scd1* deficiency enhances ATGL-mediated hydrolysis of PC and TGs, thereby releasing DHA and AA. Given that the release of phospholipid- and acylglycerol-associated DHA is essential for many of its biological activities as well as its capacity to activate nuclear receptors [39], the lipidomic analyses further provide a molecular rationale for the enrichment of genes in the DHA and PPARγ signaling pathways in *Scd1*^−/−^ naïve T cells (Fig. 3B–E, J).

### ATGL-driven hydrolysis of PC and TGs stimulates Treg differentiation in *Scd1*^−/−^ T cells by releasing DHA

**Fig. 5:**
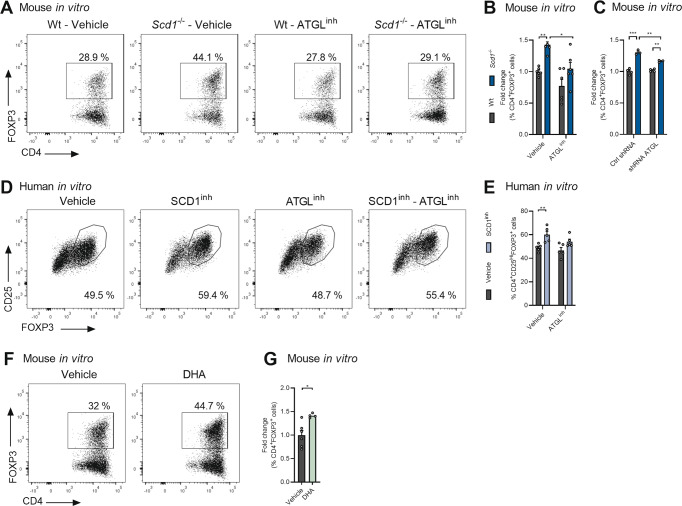
ATGL-driven release of nonesterified DHA enhances Treg differentiation in *Scd1*^−/−^ T cells. **A**–**C** Wt and *Scd1*^−/−^ mouse naïve T cells were differentiated under Treg polarizing conditions and treated with vehicle or ATGL inhibitor (ATGL^inh^, Atglistatin, 20 µM; *n* = 9 samples) or were transduced with ATGL-specific (shRNA ATGL) or control shRNA (Ctrl shRNA) (*n* = 4 samples). The frequency of CD4^+^FOXP3^+^ cells was quantified two days after Treg induction. **D**, **E** Human naïve T cells were treated with SCD1 inhibitor (SCD1^inh^, CAY10566, 1 µM), ATGL^inh^, or vehicle (*n* = 5 healthy controls). The frequency of CD25^Hi^FOXP3^+^ cells was quantified four days after Treg induction. Representative flow cytometric plots (**A**, **D**) and flow cytometric analyses (**B**, **C**, **E**) are shown. **F**, **G** Wt and *Scd1*^−/−^ mouse naïve T cells were differentiated under Treg polarizing conditions and treated with vehicle or BSA-conjugated DHA (1 µM; *n* = 4 samples). The results are pooled from at least two independent experiments and presented as the mean ± SEM. *P < 0.05; **P < 0.01; ***P < 0.001, calculated with two-tailed unpaired Student’s t-test (**B**, **C**, **E**, **G**)

Based on the RNA sequencing and lipidomic analysis, we next assessed the importance of ATGL and DHA in driving enhanced Treg differentiation of naïve *Scd1*^−/−^ T cells. By using a pharmacological inhibitor of ATGL (Atglistatin), we found that ATGL inhibition partially counteracted the enhanced induction of Tregs from *Scd1*^−/−^ naïve T cells in vitro (Fig. 5A, B) and, in parallel, markedly reduced the intracellular abundance of nonesterified DHA (Supplementary Fig. 8A). ATGL inhibition did not decrease the capacity of wt naïve T cells to differentiate into Tregs (Fig. 5A, B). Similar findings were obtained following shRNA-mediated gene silencing of *Atgl* in mouse T cells and upon exposure of human PBMC-derived naïve T cells to the ATGL inhibitor (Fig. 5C–E; Supplementary Fig. 8B). In support of a key role of DHA in driving Treg differentiation, exposure to DHA significantly promoted Treg differentiation (Fig. 5F, G). Together, these data indicate that enhanced Treg differentiation upon SCD1 inhibition and in *Scd1*^−/−^ T cells partially relies on increased ATGL activity. They further suggest that nonesterified bioactive DHA, released through ATGL-mediated hydrolysis of PC and acylglycerols, acts as an important lipid metabolite in this pathway.

### Nonesterified DHA promotes Treg differentiation through PPARγ

**Fig. 6:**
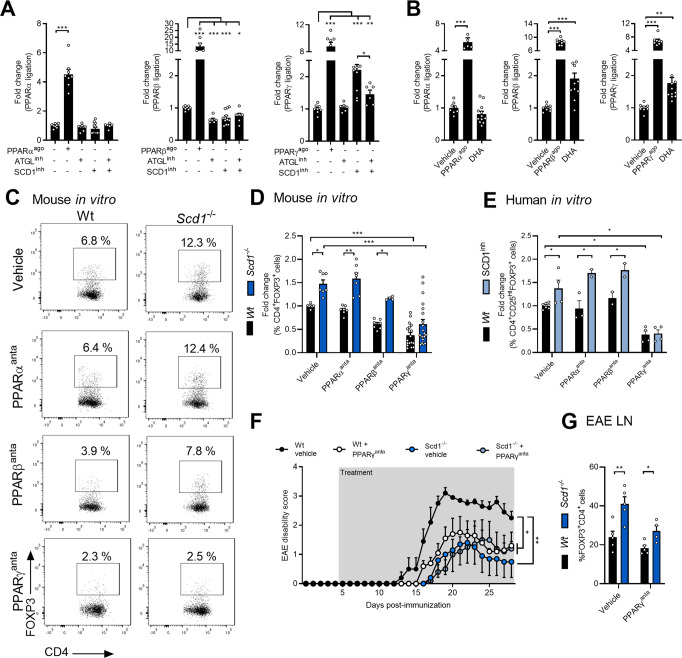
DHA-PPARγ signaling promotes Treg differentiation in the absence of SCD1. **A**, **B** Ligand-binding luciferase reporter assays were used to assess ligation of the different PPAR isoforms (*n* = 6–12 samples). Jurkat T cells were treated with vehicle, ATGL inhibitor (ATGLinh, atglistatin, 20 μM), SCD1 inhibitor (SCD1inh, CAY10566, 10 μM) (**A**), DHA (1 μM) (**B**), and/or PPAR agonists (PPARα^ago^: WY-14643, 10 μM; PPARβ^ago^: GW501516, 10 μM; PPARγ^ago^: rosiglitazone, 10 μM) for 24 h, after which PPAR ligation was assessed. **C**, **D** Wild-type (wt) and *Scd1*^−/−^ naïve T cells were differentiated under Treg polarizing conditions and treated with vehicle, PPARα antagonist (PPARα^anta^: GW6471, 25 μM; *n* = 7–13 samples), PPARβ antagonist (PPARβ^anta^: PTS58, 25 μM; *n* = 5–7 samples), and PPARγ antagonist (PPARγ^anta^: GW9662, 25 μM; *n* = 18–20 samples). The frequency of CD4^+^FOXP3^+^ cells was quantified one day after Treg induction. Representative flow cytometric plots (**C**) and flow cytometric analysis (**D**) are shown. **E** Human naïve T cells were differentiated under Treg polarizing conditions and treated with vehicle (*n* = 5 healthy controls), PPARα antagonist (PPARα^anta^: GW6471, 25 μM; *n* = 3 healthy controls), PPARβ antagonist (PPARβ^anta^: PTS58, 25 μM; *n* = 3 healthy controls) or PPARγ antagonist (PPARγ^anta^: 25 μM; *n* = 4 healthy controls). The frequency of CD25^+^FOXP3^+^ cells among total CD4^+^ cells was quantified four days after Treg induction by flow cytometry. **F**, **G** Wt and *Scd1*^*−/−*^ mice with EAE were treated daily with vehicle or a PPARγ antagonist (GW9662, 2 mg/kg, *n* = 5/group). Lymph nodes were collected 28 days postimmunization. EAE disease score (**F**) and flow cytometric analysis of the frequency of CD4^+^FOXP3^+^ cells in the lymph nodes (**G**). Data are represented as the mean ± SEM. **P < 0.01, calculated with Tukey’s post hoc analysis

Upstream analysis revealed that differentially expressed genes in *Scd1*^−/−^ naïve T cells were associated with the activation of PPARγ (Fig. 3J). Consistent with these findings, naïve *Scd1*^*−/−*^ T cells demonstrated increased expression of PPARγ-responsive genes, including *Srebpc1*, *Abca1*, *Cd36*, *Cpt1a*, and *Lpl* (Supplementary Fig. 9A). Furthermore, by using ligand-binding luciferase reporter assays, we showed that SCD1 inhibition promoted the ligation of PPARγ but not PPARα or PPARβ (Fig. 6A). Interestingly, inhibition of ATGL countered the ligation of PPARγ induced by SCD1 inhibition (Fig. 6A). In line with a key role of nonesterified DHA in the activation of PPARs, exposure to DHA induced the activation of PPARγ and PPARβ but not PPARα (Fig. 6B). Collectively, these findings strongly suggest that *Scd1* deficiency results in the ATGL-mediated release of DHA, which in turn activates PPARγ.

Given that PPARγ activation promotes Treg differentiation and that DHA is an endogenous PPARγ ligand [39–41], we next assessed the role of PPARs in driving enhanced Treg differentiation in *Scd1*^−/−^ naïve T cells. To this end, T cell cultures were exposed to well-described antagonists for the different PPAR isoforms (PPARα^anta^: WY-14643; [42, 43] PPARβ^anta^: PTS58; [44, 45] PPARγ^anta^: GW9662 [46]). Only PPARγ antagonism prevented enhanced mouse Treg differentiation induced by the absence of *Scd1* (Fig. 6C, D). Similar to murine T cells, treatment with the PPARγ antagonist reduced Treg differentiation independently of SCD1 and abolished enhanced Treg differentiation caused by SCD1 inhibition in human T cell cultures (Fig. 6E; Supplementary Fig. 9B).

Finally, to confirm the interplay between SCD1 and PPARγ in autoimmunity under physiological conditions, we exposed *Scd1*^−/−^ animals with EAE to the PPARγ antagonist GW9662. Here, we observed that *Scd1* deficiency no longer reduced EAE disease severity in the presence of GW9662 compared to that of the control animals treated with GW9662 (Fig. 6F). Surprisingly, PPARγ antagonism reduced EAE disease severity compared to that of the control mice (Fig. 6F). The latter finding contradicts the protective impact of PPARγ agonists in the EAE model [47] but is consistent with GW9662 displaying anti-inflammatory properties in in vitro immune cell cultures and an LPS-triggered acute inflammation mouse model [48]. Consistent with changes in EAE disease severity, *Scd1*^*−/−-*^induced changes in CNS inflammation, accumulation of CD3^+^ T cells, and demyelination were no longer apparent in the presence of GW9662 (Supplementary Fig. 9D–H). Furthermore, PPARγ antagonism reduced the observed increase in peripheral Treg frequency in *Scd1*^*−/−*^ animals with EAE (Fig. 6G; Supplementary Fig. 9C). These findings provide evidence that *Scd1* deficiency impacts EAE disease severity, neuroinflammation, and Treg differentiation through PPARγ under physiological conditions.

## Discussion

An imbalance between pathogenic and protective T cell subsets is considered to propel autoimmunity. The findings from this study indicate that the FA desaturase SCD1 tightly controls T cell fate and autoimmunity and that its activity is increased in CD4^+^ T cells of MS patients. We report that pharmacological inhibition and genetic deficiency of SCD1 stimulates Treg differentiation and, in parallel, attenuates neuroinflammation and disease severity in the EAE model in a T cell-dependent manner. We further provide evidence that *Scd1* deficiency promotes Treg differentiation through ATGL-dependent hydrolysis of TGs and PC, thereby releasing nonesterified bioactive DHA, a natural ligand of the nuclear receptor PPARγ. Thus, our findings identify an intricate relationship between FA desaturation, Treg differentiation, and autoimmunity.

FA metabolism is increasingly being acknowledged as a hub and driver of T cell physiology in health and disease [4, 11–18]. Our data indicate that the FA desaturase SCD1 acts as an endogenous brake on the differentiation of Tregs, thereby contributing to neuroinflammation in the EAE model. Consistent with our findings, previous studies showed that intracellular levels of substrates and products of SCD1, e.g., oleic acid (C18:1) and palmitic acid (C16:0), closely regulate T cell and Treg differentiation and maintenance [9, 13, 18]. Likewise, SCD1 inhibition was recently found to increase the frequency of follicular Tregs in the spleen following influenza immunization [49]. In contrast, *Scd1* deficiency accelerates and exacerbates the development of colitis by promoting the colitogenic potential of effector T cells [50]. Here, elevated levels of SFA increased cellular membrane fluidity and augmented the secretory profile of effector T cells. However, the authors did not assess the impact of *Scd1* deficiency on Treg physiology. Differences in the immunopathology and applied experimental animal models, *e.g*., adoptive transfer of naïve or antigen-sensitized T cells to *Rag1*^−/−^ and wt mice, respectively, might well explain the opposing outcome of *Scd1* deficiency on disease initiation and severity in the colitis and EAE model. In summary, our findings highlight the importance of fatty acid desaturation by SCD1 in controlling Treg differentiation and protective autoimmunity in the EAE model.

Our findings demonstrate that a lack of SCD1 increases ATGL-dependent hydrolysis of TGs and PC, resulting in elevated intracellular levels of nonesterified DHA, activation of PPARγ, enhanced differentiation of Tregs, and reduced EAE disease severity. Accordingly, ATGL is reported to favor the hydrolysis of TGs and phospholipids rich in PUFAs such as DHA, and DHA is a potent endogenous ligand for PPARγ [38, 51]. Furthermore, previous studies have shown that DHA and synthetic PPARγ agonists promote the differentiation and maintenance of Tregs and reduce neuroinflammation in experimental animal models [41, 52–57]. Surprisingly, we found that PPARγ antagonism reduced EAE disease severity compared to that in the control mice. The latter finding contradicts the beneficial impact of PPARγ agonists in the EAE model but is consistent with GW9662 displaying anti-inflammatory properties in vitro and in vivo [48]. Overall, our findings identify the SCD1-ATGL-PPAR axis as a key signaling pathway in driving Treg differentiation and highlight the complexity of PPAR signaling in (auto)immune disorders.

The molecular mechanisms that underlie the increased ATGL-dependent release of DHA in *Scd1*^*−/−*^ T cells remain to be clarified. In the absence of SCD1, T cells may increase ATGL-dependent intracellular release of free DHA to protect themselves against endoplasmic reticulum stress induced by elevated levels of SFAs, potentially through activation of PPARγ [58, 59]. In support of this notion, pathway analysis showed that differentially expressed genes in *Scd1*^*−/−*^ T cells were enriched in the canonical pathway ‘unfolded protein response’, a cellular response associated with ER stress [60]. Alternatively, or in parallel, changes in the activity or abundance of signal transducer and activator of transcription 5 (STAT5), SP1, insulin, and TNFα may increase ATGL expression and activity in the absence of SCD1 [61–64]. On a related note, while SFAs are generally believed to promote inflammation, our findings indicate the opposite. Lack of SCD1 enhanced Treg differentiation and suppressed neuroinflammation, despite decreasing the intracellular desaturation index. Given that PUFAs are more potent PPARγ agonists than MUFAs [51], elevated DHA levels may outweigh and nullify the inflammatory impact of SFAs in our study. Alternatively, while the majority of studies determined the effects of exogenous SFAs on T cell physiology, the mode of action and cellular outcome of exogenously and endogenously altered SFA levels might differ [65]. Collectively, our findings indicate that the ATGL-DHA-PPARγ signaling axis is essential in driving enhanced Treg differentiation in *Scd1*-deficient T cells. However, more research is warranted to define the molecular mechanisms that control the activity of the ATGL-DHA-PPARγ axis.

LC‒ESI‒MS/MS analysis demonstrated that a lack of SCD1 decreases the bulk of major fatty acid-containing lipid species in T cells without affecting cell size and granularity. Given that differentially expressed genes in *Scd1*^−/−^ naïve T cells were enriched in pathways related to lipid efflux, these findings may reflect an increased capacity of *Scd1*^−/−^ T cells to dispose intracellular cholesterol and phospholipids. A complementary reduction in lipogenesis and activation in lipolysis may contribute to the observed decreased lipid load in *Scd1*^−/−^ T cells [66]. Alongside elevated intracellular levels of nonesterified DHA, LC‒MS/MS analysis revealed an increased abundance of the ω6 PUFA AA (C20:4) and its derivatives 11-HETE and 15-HETE in *Scd1*^−/−^ naïve T cells. Given that ATGL preferentially hydrolyses lipid species rich in PUFAs such as DHA and AA, these findings are consistent with increased ATGL activity in *Scd1*^−/−^ naïve T cells. Of interest, previous studies demonstrated that AA and its derivatives are endogenous PPARγ agonists and closely associated with Treg differentiation [51, 67, 68]. To what extent AA, 11-HETE, and 15-HETE contribute to enhanced Treg differentiation in *Scd1*^−/−^ T cells remains to be determined.

A growing body of epidemiological, preclinical, and observational studies suggests that obesity and Western diets rich in saturated fats, cholesterol, and carbohydrates are associated with Treg dysfunction, neuroinflammation, and autoimmunity [5, 6, 69–71]. Of interest, obesity and consumption of a Western diet result in markedly elevated expression and activity of hepatic SCD1 [72–74], and *Scd1*-deficient mice are protected from diet-induced obesity [75, 76]. Hence, our findings argue that SCD1 is an enzymatic driver underlying the impact of obesity and Western-type diets on immune cell imbalances and disease progression in autoimmune disorders such as MS. Vice versa, given that DHA inhibits *Scd1* expression [77], the beneficial impact of DHA supplementation on Treg differentiation, EAE severity, and disease pathology of autoimmune disorders such as MS, systemic lupus erythematosus, and rheumatoid arthritis might depend on changes in SCD1 activity [54, 78]. Future dietary studies are warranted to unravel the causal role of SCD1 in driving the beneficial and detrimental impact of DHA and a Western diet, respectively, on the immunopathology of MS and other autoimmune disorders.

In summary, we report that SCD1 plays a crucial role in autoimmunity by suppressing Treg differentiation. Conclusions from this study extend our previous findings that SCD1 impairs the reparative properties of macrophages and microglia in the CNS [26]. Together, our findings argue that pharmacological inhibitors of SCD1 are a promising therapeutic strategy to simultaneously suppress autoimmunity, reduce neuroinflammation, and promote CNS repair. Our findings further provide a potential molecular rationale for the beneficial and detrimental impact of dietary factors on autoimmunity and validate other recent studies that identified changes in fatty acid metabolism as a crucial determinant of the pathological outcome of these factors. Finally, this study endorses the essential role of PPARγ in driving autoimmunity and disease progression in MS [79–83]. Altogether, our findings place SCD1 at the crossroads of autoimmunity and lipid metabolism.

## Supplementary information

Supplementary Table 1: LC-MS/MS parameters

Supplementary Table 2: Differentially expressed genes in Scd1-deficient CD4+ naïve T cells vs. wt CD4+ naïve T cells

Supplementary Table 3: Differentially expressed genes in Scd1-deficient CD4+ early iTregs vs. wt CD4+ early iTregs

Supplemental Figures Descriptions

Supplemental Figure 1

Supplemental Figure 2

Supplemental Figure 3

Supplemental Figure 4

Supplemental Figure 5

Supplemental Figure 6

Supplemental Figure 7

Supplemental Figure 8

Supplemental Figure 9
