# Supplementary material for: Fatty acid desaturation by stearoyl-CoA desaturase-1 controls regulatory T cell differentiation and autoimmunity
Source: Cell Mol Immunol. 2023 Apr 12;20(6):666–79. doi: 10.1038/s41423-023-01011-2 (PMC10229556; doi:10.1038/s41423-023-01011-2)
Supplement: Supplementary file 1 — Supplementary Table 1: LC-MS/MS parameters [file 41423_2023_1011_MOESM1_ESM.docx]

**Supplementary Table 1: LC-MS/MS parameters**

| **Group** | **Compound** | **Lipid Maps ID** | **Retention time [min]** | **m/z in Q1** | **m/z in Q3** | **Declustering potential [V]** | **Collision energy [V]** | **Collision cell exit potential [V]** |
| --- | --- | --- | --- | --- | --- | --- | --- | --- |
| Epoxyeicosatrienoic acids (EET) | 14(15)-EET | LMFA03080005 | 8.1 | 319 | 218.9 | -5 | -16 | -55 |
| Hydroxydocosahexaenoic acids (HDHA) | 14(S)-HDHA | LMFA04000058 | 8 | 343.1 | 204.9 | -60 | -18 | -27 |
| Hydroxydocosahexaenoic acids (HDHA) | 17-HDHA | LMFA04000072 | 7.9 | 343.1 | 245 | -65 | -16 | -15 |
| Hydroxyeicosapentaenoic acids (HEPE) | 12-HEPE | LMFA03070031 | 7.6 | 317 | 179 | -60 | -18 | -17 |
| Hydroxyeicosapentaenoic acids (HEPE) | 15-HEPE | LMFA03070009 | 7.5 | 317.1 | 219 | -65 | -18 | -19 |
| Hydroxyeicosatetraenoic acids (HETE) | 11-HETE | LMFA03060003 | 7.9 | 319.1 | 167 | -70 | -22 | -15 |
| Hydroxyeicosatetraenoic acids (HETE) | 12-HETE | LMFA03060007 | 7.9 | 319.1 | 179 | -65 | -20 | -23 |
| Hydroxyeicosatetraenoic acids (HETE) | 15-HETE | LMFA03060001 | 7.8 | 319.1 | 219.1 | -55 | -18 | -9 |
| Hydroxyeicosatetraenoic acids (HETE) | 17-OH-DH-HETE | N/A | 8.2 | 347.1 | 247 | -110 | -22 | -27 |
| Hydroxyeicosatetraenoic acids (HETE) | 5-HETE | LMFA03060002 | 8 | 319.1 | 115 | -65 | -18 | -11 |
| Hydroxyeicosatetraenoic acids (HETE) | 8-HETE | LMFA03060006 | 7.9 | 319.1 | 154.9 | -70 | -20 | -19 |
| Hydroxyoctadecadienoic acids (HoDE) | 13-HoDE | LMFA02000228 | 7.7 | 295 | 194.9 | -110 | -24 | -21 |
| Hydroxyoctadecadienoic acids (HoDE) | 9-HoDE | LMFA02000188 | 7.7 | 295 | 171 | -130 | -22 | -7 |
| Hydroxyoctadecatrienoic acids (HoTrE) | 9-HoTrE | LMFA02000024 | 7.4 | 293 | 170.9 | -75 | -20 | -15 |
| Internal standards | 15-HETE-d8 | LMFA03060080 | 7.8 | 327.2 | 226 | -85 | -18 | -11 |
| Internal standards | DHA-d5 | LMFA01030762 | 8.8 | 332 | 288.1 | -75 | -16 | -13 |
| Internal standards | LTB4-d4 | LMFA03020030 | 6.9 | 339.1 | 196.9 | -70 | -22 | -19 |
| Internal standards | PGE2-d4 | LMFA03010008 | 4.9 | 355.1 | 193 | -50 | -26 | -17 |
| Keto-eicosatetraenoic acids (KETE/OxoETE) | 12-KETE | LMFA03060019 | 7.9 | 317 | 153 | -60 | -22 | -9 |
| Keto-eicosatetraenoic acids (KETE/OxoETE) | 15-KETE | LMFA03060051 | 7.8 | 317 | 113 | -10 | -22 | -5 |
| Keto-eicosatetraenoic acids (KETE/OxoETE) | 5-KETE | LMFA03060011 | 8.1 | 317 | 203.1 | -70 | -24 | -11 |
| Leukotrienes (LT) | 6-trans-LTB4 | LMFA03020013 | 6.7 | 335.1 | 194.9 | -105 | -22 | -11 |
| Polyunsaturated fatty acids | AA | LMFA01030001 | 8.8 | 303 | 205.1 | -155 | -20 | -11 |
| Polyunsaturated fatty acids | AdA | LMFA01030178 | 9.1 | 331.1 | 233 | -130 | -22 | -11 |
| Polyunsaturated fatty acids | ALA/GLA | LMFA01030152 / LMFA01030141 | 8.6 | 277 | 233 | -90 | -22 | -29 |
| Polyunsaturated fatty acids | DGLA | LMFA01030158 | 9 | 305.1 | 261.2 | -85 | -22 | -13 |
| Polyunsaturated fatty acids | DHA | LMFA01030185 | 8.8 | 327.1 | 229.2 | -115 | -18 | -11 |
| Polyunsaturated fatty acids | DPAn-3 | LMFA04000044 | 8.9 | 329.1 | 231.1 | -50 | -20 | -17 |
| Polyunsaturated fatty acids | DPAn-6 | LMFA01030182 | 9 | 329.1 | 231.1 | -50 | -20 | -17 |
| Polyunsaturated fatty acids | EPA | LMFA01030759 | 8.6 | 301 | 202.9 | -125 | -18 | -21 |
| Polyunsaturated fatty acids | LA | LMFA01030120 | 8.8 | 279 | 261 | -115 | -28 | -13 |
| Prostaglandins (PG) | 15-deoxy-PGJ2 | LMFA03010021 | 7.3 | 315 | 203 | -50 | -28 | -19 |
| Prostaglandins (PG) | 15-keto-PGE2 | LMFA03010030 | 4.5 | 349 | 234.9 | -65 | -20 | -13 |
| Prostaglandins (PG) | PGD2 | LMFA03010004 | 5 | 351.1 | 233 | -30 | -16 | -13 |
| Prostaglandins (PG) | PGE2 | LMFA03010003 | 4.9 | 351.2 | 271.1 | -50 | -22 | -21 |
| Prostaglandins (PG) | PGF2alpha | LMFA03010002 | 5.2 | 353.1 | 193 | -80 | -34 | -11 |
| Prostaglandins (PG) | PGJ2 | LMFA03010019 | 6.1 | 333 | 271 | -30 | -22 | -17 |
| Thromboxanes (Tx) | TXB2 | LMFA03030002 | 4.6 | 369.1 | 169 | -55 | -24 | -15 |
