# Supplementary material for: Fatty acid desaturation by stearoyl-CoA desaturase-1 controls regulatory T cell differentiation and autoimmunity
Source: Cell Mol Immunol. 2023 Apr 12;20(6):666–79. doi: 10.1038/s41423-023-01011-2 (PMC10229556; doi:10.1038/s41423-023-01011-2)
Supplement: Supplementary file 3 — Supplementary Table 3: Differentially expressed genes in Scd1-deficient CD4+ early iTregs vs. wt CD4+ early iTregs [file 41423_2023_1011_MOESM3_ESM.docx]

**Supplementary Table 3: Differentially expressed genes in *Scd1*-deficient CD4^+^ early iTregs vs. wt CD4^+^ early iTregs**

| **Upregulated genes** | | | | | |
| --- | --- | --- | --- | --- | --- |
| **Symbol** | **Entrez Gene Name** | **Basemean** | **Log2(Fold change)** | **P value** | **Padj** |
| *Hnrnpa3* | heterogeneous nuclear ribonucleoprotein A3 | 434.321 | 0,926692 | 0,0018069 | 1 |
| *Cct6a* | chaperonin containing Tcp1, subunit 6a (zeta) | 916.204 | 0,763597 | 0,0020737 | 1 |
| *Eef1a1* | eukaryotic translation elongation factor 1 alpha 1 | 383.566 | 0,767301 | 0,0021950 | 1 |
| *S1pr2* | sphingosine-1-phosphate receptor 2 | 788.193 | 2,197226 | 0,0029230 | 1 |
| *Smg7* | SMG7 nonsense mediated mRNA decay factor | 833.553 | 0,663588 | 0,0036513 | 1 |
| *Zfp608* | zinc finger protein 608 | 231.758 | 3,869541 | 0,0060571 | 1 |
| *Taf4a* | TATA-Box Binding Protein Associated Factor 4 | 149.316 | 1,480840 | 0,0065072 | 1 |
| *Il12rb1* | interleukin 12 receptor, beta 1 | 52.922 | 2,928081 | 0,0066381 | 1 |
| *Tmtc4* | transmembrane and tetratricopeptide repeat containing 4 | 143.735 | 1,585436 | 0,0070018 | 1 |
| *Pogz* | pogo transposable element with ZNF domain | 327.535 | 0,875648 | 0,0071574 | 1 |
| *A330017A19Rik* | RIKEN cDNA A330017A19 gene | 385.459 | 2,753858 | 0,0091918 | 1 |
| *Alkbh8* | alkB homolog 8, tRNA methyltransferase | 247.570 | 1,014750 | 0,0097701 | 1 |
| *Lrrc32* | leucine rich repeat containing 32 | 518.749 | 2,230286 | 0,0100465 | 1 |
| *Senp1* | SUMO1/sentrin specific peptidase 1 | 558.589 | 0,750854 | 0,0103650 | 1 |
| *Blmh* | bleomycin hydrolase | 673.579 | 0,717974 | 0,0108972 | 1 |
| *Pom121* | nuclear pore membrane protein 121 | 40.431 | 0,739697 | 0,0112915 | 1 |
| *Preb* | prolactin regulatory element binding | 257.575 | 0,993610 | 0,0120614 | 1 |
| *Rpl14-ps1* | ribosomal protein L14, pseudogene 1 | 184.731 | 0,464125 | 0,0126917 | 1 |
| *Il1rl2* | interleukin 1 receptor-like 2 | 511.871 | 2,136402 | 0,0133403 | 1 |
| *Ulk2* | unc-51 like kinase 2 | 106.488 | 1,404372 | 0,0139800 | 1 |
| *Pdia3* | protein disulfide isomerase associated 3 | 329.291 | 0,422689 | 0,0139923 | 1 |
| *Gm12791* | predicted gene 12791 | 100.855 | 1,419500 | 0,0139947 | 1 |
| *Gm6565* | predicted gene 6565 | 179.594 | 1,144424 | 0,0144412 | 1 |
| *Maff* | v-maf musculoaponeurotic fibrosarcoma oncogene family, protein F (avian) | 128.358 | 1,250644 | 0,0146926 | 1 |
| *Arl14ep* | ADP-ribosylation factor-like 14 effector protein | 121.003 | 1,364151 | 0,0148125 | 1 |
| *Cdv3* | carnitine deficiency-associated gene expressed in ventricle 3 | 695.970 | 0,648626 | 0,0149101 | 1 |
| *Ddx43* | DEAD box helicase 43 | 26.590 | 3,137235 | 0,0150452 | 1 |
| *Ctsb* | cathepsin B | 688.920 | 0,766183 | 0,0151727 | 1 |
| *Pgpep1l* | pyroglutamyl-peptidase I-like | 71.181 | 1,732893 | 0,0153662 | 1 |
| *Hectd2* | HECT domain E3 ubiquitin protein ligase 2 | 709.947 | 1,652526 | 0,0154759 | 1 |
| *Dstn* | destrin | 25.307 | 0,897141 | 0,0158495 | 1 |
| *Pvt1* | Pvt1 oncogene | 610.973 | 1,741607 | 0,0169685 | 1 |
| *Cd63* | CD63 antigen | 592.648 | 2,527900 | 0,0176099 | 1 |
| *Iigp1* | interferon inducible GTPase 1 | 227.260 | 0,116342 | 0,0177471 | 1 |
| *Rnmt* | RNA (guanine-7-) methyltransferase | 434.540 | 0,720381 | 0,0177749 | 1 |
| *P2ry10* | purinergic receptor P2Y, G-protein coupled 10 | 171.309 | 1,055188 | 0,0193415 | 1 |
| *Tacc2* | transforming, acidic coiled-coil containing protein 2 | 274.096 | 0,812408 | 0,0203003 | 1 |
| *Dnajb12* | DnaJ heat shock protein family (Hsp40) member B12 | 223.467 | 0,867907 | 0,0209224 | 1 |
| *Cep57l1* | centrosomal protein 57-like 1 | 919.341 | 1,414499 | 0,0213860 | 1 |
| *Id2* | inhibitor of DNA binding 2 | 101.540 | 0,592708 | 0,0217762 | 1 |
| *Casp8ap2* | caspase 8 associated protein 2 | 721.643 | 0,547294 | 0,0218211 | 1 |
| *Usp14* | ubiquitin specific peptidase 14 | 635.399 | 0,661642 | 0,0223327 | 1 |
| *Lgalsl* | galectin like | 246.054 | 0,304476 | 0,0235138 | 1 |
| *Gnptg* | N-acetylglucosamine-1-phosphotransferase, gamma subunit | 14.102 | 1,058726 | 0,0238458 | 1 |
| *Slc39a8* | solute carrier family 39 (metal ion transporter), member 8 | 156.383 | 1,186857 | 0,0248249 | 1 |
| *Gm8822* | predicted gene 8822 | 955.356 | 1,391440 | 0,0249014 | 1 |
| *Lrrc20* | leucine rich repeat containing 20 | 561.621 | 1,752091 | 0,0256319 | 1 |
| *Ctnna1* | catenin (cadherin associated protein), alpha 1 | 302.628 | 0,761410 | 0,0259586 | 1 |
| *Mtmr6* | myotubularin related protein 6 | 184.340 | 1,034348 | 0,0259715 | 1 |
| *Tgfbr3* | transforming growth factor, beta receptor III | 136.980 | 1,071523 | 0,0263245 | 1 |
| *Rbpj* | recombination signal binding protein for immunoglobulin kappa J region | 150.010 | 0,427990 | 0,0274775 | 1 |
| *Prkar2a* | protein kinase, cAMP dependent regulatory, type II alpha | 600.226 | 0,632786 | 0,0274849 | 1 |
| *Aim2* | absent in melanoma 2 | 256.271 | 0,967345 | 0,0278640 | 1 |
| *P4ha1* | procollagen-proline, 2-oxoglutarate 4-dioxygenase (proline 4-hydroxylase), alpha 1 polypeptide | 191.379 | 0,918925 | 0,0294170 | 1 |
| *Gpr162* | G protein-coupled receptor 162 | 151.173 | 3,344225 | 0,0301264 | 1 |
| *Cep83* | centrosomal protein 83 | 784.290 | 0,563279 | 0,0302946 | 1 |
| *Gm8337* | predicted gene 8337 | 43.914 | 2,028164 | 0,0308223 | 1 |
| *Kdelc1* | protein O-Glucosyltransferase 2 | 242.316 | 2,779511 | 0,0321882 | 1 |
| *Gm10698* | predicted gene 10698 | 203.236 | 2,997744 | 0,0323362 | 1 |
| *Hexim1* | hexamethylene bis-acetamide inducible 1 | 509.429 | 0,547309 | 0,0323839 | 1 |
| *Gm18889* | predicted gene, 18889 | 427.956 | 1,855917 | 0,0325256 | 1 |
| *Wdr75* | WD repeat domain 75 | 100.919 | 0,434442 | 0,0328431 | 1 |
| *Zfp52* | zinc finger protein 52 | 121.963 | 1,160756 | 0,0333214 | 1 |
| *Gm12096* | predicted gene 12096 | 171.880 | 0,335533 | 0,0341805 | 1 |
| *Arpc3* | actin related protein 2/3 complex, subunit 3 | 158.826 | 0,405178 | 0,0342091 | 1 |
| *Gm2223* | predicted pseudogene 2223 | 364.045 | 0,690463 | 0,0342222 | 1 |
| *Celf1* | CUGBP, Elav-like family member 1 | 223.209 | 0,344077 | 0,0350707 | 1 |
| *Gm22009* | predicted gene, 22009 | 395.078 | 2,250878 | 0,0350904 | 1 |
| *Papss1* | 3'-phosphoadenosine 5'-phosphosulfate synthase 1 | 188.117 | 0,833743 | 0,0361784 | 1 |
| *Zfp518b* | zinc finger protein 518B | 119.037 | 3,734110 | 0,0374531 | 1 |
| *Zfp623* | zinc finger protein 623 | 897.663 | 1,354521 | 0,0383157 | 1 |
| *Spp1* | secreted phosphoprotein 1 | 366.937 | 0,268774 | 0,0389632 | 1 |
| *Hsd17b12* | hydroxysteroid (17-beta) dehydrogenase 12 | 651.706 | 0,543610 | 0,0393151 | 1 |
| *Skp2* | S-phase kinase-associated protein 2 | 573.563 | 1,760089 | 0,0394093 | 1 |
| *Pid1* | phosphotyrosine interaction domain containing 1 | 118.700 | 3,759140 | 0,0395701 | 1 |
| *Sec63* | SEC63-like (S, cerevisiae) | 700.403 | 0,497083 | 0,0400926 | 1 |
| *Cdc42bpb* | CDC42 binding protein kinase beta | 158.441 | 3,224391 | 0,0407281 | 1 |
| *Rngtt* | RNA guanylyltransferase and 5'-phosphatase | 346.918 | 0,675525 | 0,0411199 | 1 |
| *Hspa13* | heat shock protein 70 family, member 13 | 106.992 | 1,123288 | 0,0413384 | 1 |
| *Amn1* | antagonist of mitotic exit network 1 | 49.080 | 1,637226 | 0,0418306 | 1 |
| *Marveld1* | MARVEL (membrane-associating) domain containing 1 | 10.979 | 1,148840 | 0,0420751 | 1 |
| *Zfp955b* | zinc finger protein 955B | 212.732 | 2,868317 | 0,0421133 | 1 |
| *Atad5* | ATPase family, AAA domain containing 5 | 138.901 | 1,195913 | 0,0422371 | 1 |
| *Braf* | Braf transforming gene | 187.295 | 0,940970 | 0,0424681 | 1 |
| *Lyz2* | lysozyme 2 | 103.593 | 1,749370 | 0,0425077 | 1 |
| *3830406C13Rik* | RIKEN cDNA 3830406C13 gene | 177.821 | 0,881889 | 0,0441261 | 1 |
| *Rpp25l* | ribonuclease P/MRP 25 subunit-like | 241.162 | 0,790300 | 0,0443439 | 1 |
| *Oxct1* | 3-oxoacid CoA transferase 1 | 101.581 | 0,451065 | 0,0446444 | 1 |
| *Txlna* | taxilin alpha | 81.708 | 0,446438 | 0,0450186 | 1 |
| *Gm7722* | predicted gene 7722 | 204.312 | 2,828058 | 0,0462389 | 1 |
| *Txnl1* | thioredoxin-like 1 | 109.368 | 0,414637 | 0,0466168 | 1 |
| *Pus10* | pseudouridylate synthase 10 | 104.822 | 1,120842 | 0,0467276 | 1 |
| *Pabpc1* | poly(A) binding protein, cytoplasmic 1 | 256.302 | 0,375106 | 0,0467432 | 1 |
| *Tmpo* | thymopoietin | 920.178 | 0,428700 | 0,0470556 | 1 |
| *Ap3b1* | adaptor-related protein complex 3, beta 1 subunit | 626.519 | 0,479017 | 0,0480137 | 1 |
| *Plekha1* | pleckstrin homology domain containing, family A (phosphoinositide binding specific) member 1 | 320.788 | 0,668014 | 0,0485230 | 1 |
| **Downregulated genes** | | | | | |
| **Symbol** | **Entrez Gene Name** | **Basemean** | **Log2(Fold change)** | **P value** | **Padj** |
| *Tm9sf1* | transmembrane 9 superfamily member 1 | 324.695 | -4,339 | 0,00109 | 1 |
| *Ssbp3* | single-stranded DNA binding protein 3 | 577.093 | -0,805 | 0,00312 | 1 |
| *Gm12251* | predicted gene 12251 | 553.657 | -2,201 | 0,00532 | 1 |
| *Sash3* | SAM and SH3 domain containing 3 | 567.953 | -0,804 | 0,00642 | 1 |
| *Gm5453* | predicted gene 5453 | 120.426 | -1,676 | 0,00702 | 1 |
| *Lysmd4* | LysM, putative peptidoglycan-binding, domain containing 4 | 47.390 | -2,427 | 0,00811 | 1 |
| *B130006D01Rik* | RIKEN cDNA B130006D01 gene | 369.860 | -3,047 | 0,00978 | 1 |
| *Thumpd2* | THUMP domain containing 2 | 605.892 | -2,114 | 0,01010 | 1 |
| *Tmem80* | transmembrane protein 80 | 109.380 | -1,479 | 0,01088 | 1 |
| *Gm6030* | predicted gene 6030 | 204.157 | -0,471 | 0,01157 | 1 |
| *Ccdc62* | coiled-coil domain containing 62 | 16.820 | -4,153 | 0,01192 | 1 |
| *Fam20a* | FAM20A, golgi associated secretory pathway pseudokinase | 254.729 | -3,967 | 0,01202 | 1 |
| *Ubfd1* | ubiquitin family domain containing 1 | 28.076 | -0,873 | 0,01260 | 1 |
| *Gm17066* | predicted gene 17066 | 224.652 | -0,983 | 0,01283 | 1 |
| *Htra2* | HtrA serine peptidase 2 | 160.878 | -1,213 | 0,01353 | 1 |
| *Tmem106b* | transmembrane protein 106B | 316.559 | -0,988 | 0,01364 | 1 |
| *Paip2* | polyadenylate-binding protein-interacting protein 2 | 133.231 | -0,510 | 0,01398 | 1 |
| *Aup1* | ancient ubiquitous protein 1 | 601.800 | -0,759 | 0,01402 | 1 |
| *Tomm5* | translocase of outer mitochondrial membrane 5 | 148.068 | -0,118 | 0,01407 | 1 |
| *Zfp386* | zinc finger protein 386 (Kruppel-like) | 15.670 | -1,401 | 0,01415 | 1 |
| *Zfp287* | zinc finger protein 287 | 763.133 | -1,737 | 0,01650 | 1 |
| *Nadk2* | NAD kinase 2, mitochondrial | 487.261 | -2,423 | 0,01691 | 1 |
| *Acrbp* | proacrosin binding protein | 853.465 | -1,532 | 0,01758 | 1 |
| *Pms1* | PMS1 homolog 1, mismatch repair system component | 109.106 | -1,289 | 0,01764 | 1 |
| *Dcaf12* | DDB1 and CUL4 associated factor 12 | 2.810 | -0,869 | 0,01774 | 1 |
| *Acot2* | acyl-CoA thioesterase 2 | 271.926 | -0,907 | 0,01915 | 1 |
| *Cog2* | component of oligomeric golgi complex 2 | 425.366 | -0,813 | 0,01930 | 1 |
| *Eya3* | EYA transcriptional coactivator and phosphatase 3 | 296.648 | -0,786 | 0,01957 | 1 |
| *Zfp867* | zinc finger protein 867 | 296.631 | -2,770 | 0,02118 | 1 |
| *Bend7* | BEN domain containing 7 | 146.397 | -0,394 | 0,02121 | 1 |
| *Sgpp1* | sphingosine-1-phosphate phosphatase 1 | 174.073 | -0,985 | 0,02145 | 1 |
| *Golph3l* | golgi phosphoprotein 3-like | 198.533 | -0,962 | 0,02148 | 1 |
| *Trim34a* | tripartite motif-containing 34A | 876.880 | -1,844 | 0,02335 | 1 |
| *Rnaseh2a* | ribonuclease H2, large subunit | 200.285 | -0,971 | 0,02351 | 1 |
| *Apba3* | amyloid beta (A4) precursor protein-binding, family A, member 3 | 135.872 | -1,137 | 0,02356 | 1 |
| *Atp5s* | ATP synthase interacting protein 5 | 207.372 | -3,650 | 0,02359 | 1 |
| *Myl12b* | myosin, light chain 12B, regulatory | 479.058 | -0,664 | 0,02380 | 1 |
| *Abhd1* | abhydrolase domain containing 1 | 286.048 | -3,347 | 0,02438 | 1 |
| *Twsg1* | twisted gastrulation BMP signaling modulator 1 | 464.425 | -0,687 | 0,02549 | 1 |
| *Fam65a* | FAM65A, golgi associated secretory pathway pseudokinase | 173.570 | -1,310 | 0,02556 | 1 |
| *Pbx2* | pre B cell leukemia homeobox 2 | 293.319 | -0,821 | 0,02578 | 1 |
| *Itfg1* | integrin alpha FG-GAP repeat containing 1( | 118.946 | -1,304 | 0,02642 | 1 |
| *Gm11343* | predicted gene 11343 | 186.657 | -3,457 | 0,02671 | 1 |
| *Mtmr14* | myotubularin related protein 14 | 126.588 | -0,115 | 0,02803 | 1 |
| *Cmtr2* | cap methyltransferase 2 | 944.351 | -1,294 | 0,02846 | 1 |
| *Tmem63b* | transmembrane protein 63b | 10.899 | -1,237 | 0,02861 | 1 |
| *G6pdx* | glucose-6-phosphate dehydrogenase X-linked | 144.454 | -1,087 | 0,02866 | 1 |
| *Phip* | pleckstrin homology domain interacting protein | 713.377 | -0,726 | 0,02883 | 1 |
| *Flcn* | folliculin | 121.614 | -1,406 | 0,02921 | 1 |
| *Abtb1* | ankyrin repeat and BTB (POZ) domain containing 1 | 107.689 | -1,304 | 0,02925 | 1 |
| *Cc2d1a* | coiled-coil and C2 domain containing 1A | 631.567 | -1,563 | 0,02937 | 1 |
| *Dapk1* | death associated protein kinase 1 | 112.684 | -1,669 | 0,02958 | 1 |
| *Pink1* | PTEN induced putative kinase 1 | 387.614 | -1,045 | 0,02973 | 1 |
| *Zcchc9* | zinc finger, CCHC domain containing 9 | 513.519 | -0,622 | 0,03003 | 1 |
| *Rps23* | ribosomal protein S23 | 100.632 | -0,594 | 0,03056 | 1 |
| *Trappc5* | trafficking protein particle complex 5 | 337.714 | -0,674 | 0,03088 | 1 |
| *Rpl31-ps12* | ribosomal protein L31, pseudogene 1 2 | 127.387 | -3,749 | 0,03242 | 1 |
| *Evi5* | ecotropic viral integration site 5 | 347.519 | -2,215 | 0,03301 | 1 |
| *Trip6* | thyroid hormone receptor interactor 6 | 120.172 | -0,370 | 0,03317 | 1 |
| *Rnf43* | ring finger protein 43 | 173.104 | -3,389 | 0,03343 | 1 |
| *Acss1* | acyl-CoA synthetase short-chain family member 1 | 872.084 | -1,730 | 0,03458 | 1 |
| *Gse1* | genetic suppressor element 1, coiled-coil protein | 235.122 | -0,768 | 0,03460 | 1 |
| *Cdc42ep3* | CDC42 effector protein (Rho GTPase binding) 3 | 846.060 | -1,418 | 0,03509 | 1 |
| *Gm11836* | predicted gene 11836 | 271.414 | -2,613 | 0,03622 | 1 |
| *Kcnmb4* | potassium large conductance calcium-activated channel, subfamily M, beta member 4 | 13.033 | -1,507 | 0,03719 | 1 |
| *Cuedc1* | CUE domain containing 1 | 327.491 | -2,452 | 0,03727 | 1 |
| *Gprasp1* | G protein-coupled receptor associated sorting protein 1 | 182.513 | -0,932 | 0,03802 | 1 |
| *Clta* | clathrin, light polypeptide (Lca) | 128.379 | -0,430 | 0,03859 | 1 |
| *Sertad3* | SERTA domain containing 3 | 698.999 | -1,548 | 0,03903 | 1 |
| *B230216N24Rik* | RIKEN cDNA B230216N24 gene | 111.526 | -3,607 | 0,03946 | 1 |
| *Mapk14* | mitogen-activated protein kinase 14 | 369.585 | -0,675 | 0,03954 | 1 |
| *Rpl18a* | ribosomal protein L18A | 764.281 | -0,609 | 0,03954 | 1 |
| *Gm9828* | predicted gene 9828 | 151.604 | -3,230 | 0,03974 | 1 |
| *Gm5576* | predicted pseudogene 5576 | 860.688 | -1,313 | 0,03995 | 1 |
| *Coro1a* | coronin, actin binding protein 1A | 165.708 | -0,467 | 0,04014 | 1 |
| *Rras2* | related RAS viral (r-ras) oncogene 2 | 359.787 | -0,686 | 0,04017 | 1 |
| *Apoo* | apolipoprotein O | 594.961 | -1,712 | 0,04025 | 1 |
| *Orai2* | ORAI calcium release-activated calcium modulator 2 | 741.756 | -1,336 | 0,04063 | 1 |
| *Bptf* | bromodomain PHD finger transcription factor | 247.318 | -0,382 | 0,04185 | 1 |
| *Wbscr16* | regulator of chromosome condensation RCC1L | 179.381 | -0,938 | 0,04246 | 1 |
| *Gm4613* | predicted gene 4613 | 677.748 | -1,404 | 0,04269 | 1 |
| *Tpcn1* | two pore channel 1 | 107.273 | -1,304 | 0,04307 | 1 |
| *Acat1* | acetyl-Coenzyme A acetyltransferase 1 | 311.102 | -0,656 | 0,04308 | 1 |
| *Fam78a* | family with sequence similarity 78, member A | 607.760 | -0,888 | 0,04330 | 1 |
| *Gm11927* | predicted gene 11927 | 283.133 | -0,276 | 0,04346 | 1 |
| *Dcaf11* | DDB1 and CUL4 associated factor 11 | 329.016 | -0,704 | 0,04406 | 1 |
| *Spns1* | SPNS lysolipid transporter 1, lysophospholipid | 109.329 | -1,072 | 0,04426 | 1 |
| *Ypel3* | yippee like 3 | 738.014 | -0,573 | 0,04435 | 1 |
| *Snhg6* | small nucleolar RNA host gene 6 | 284.715 | -0,774 | 0,04437 | 1 |
| *Acp5* | acid phosphatase 5, tartrate resistant | 313.738 | -1,052 | 0,04506 | 1 |
| *Hmha1* | minor histocompatibility protein HA-1 | 187.530 | -0,580 | 0,04514 | 1 |
| *Pdp1* | pyruvate dehydrogenase phosphatase catalytic subunit 1 | 259.191 | -2,512 | 0,04546 | 1 |
| *Wwp2* | WW domain containing E3 ubiquitin protein ligase 2 | 341.335 | -0,734 | 0,04591 | 1 |
| *Mtr* | 5-methyltetrahydrofolate-homocysteine methyltransferase | 173.346 | -0,851 | 0,04631 | 1 |
| *Twf2* | twinfilin actin binding protein 2 | 445.433 | -0,580 | 0,04666 | 1 |
| *Gm13378* | predicted gene 13378 | 116.548 | -1,074 | 0,04673 | 1 |
| *Mfsd12* | major facilitator superfamily domain containing 12 | 831.681 | -1,316 | 0,04679 | 1 |
| *Mphosph8* | M-phase phosphoprotein 8 | 134.399 | -0,449 | 0,04682 | 1 |
| *Letmd1* | LETM1 domain containing 1 | 237.315 | -0,729 | 0,04697 | 1 |
| *Sdf2* | stromal cell derived factor 2 | 332.500 | -0,620 | 0,04724 | 1 |
| *Vangl2* | VANGL planar cell polarity 2 | 49.755 | -1,703 | 0,04789 | 1 |
| *Elf1* | E74 like ETS transcription factor 1 | 117.855 | -0,473 | 0,04813 | 1 |
| *Brd4* | bromodomain containing 4 | 129.604 | -0,386 | 0,04845 | 1 |
| *Slc25a53* | solute carrier family 25, member 53 | 389.908 | -2,158 | 0,04845 | 1 |
| *Ubash3a* | ubiquitin associated and SH3 domain containing, A | 251.040 | -0,731 | 0,04890 | 1 |
| *Rbm39* | RNA binding motif protein 39 | 4.094 | -0,310 | 0,04923 | 1 |
| *Iqcc* | IQ motif containing C | 753.413 | -1,393 | 0,04971 | 1 |
