# Supplementary material for: Fatty acid desaturation by stearoyl-CoA desaturase-1 controls regulatory T cell differentiation and autoimmunity
Source: Cell Mol Immunol. 2023 Apr 12;20(6):666–79. doi: 10.1038/s41423-023-01011-2 (PMC10229556; doi:10.1038/s41423-023-01011-2)
Supplement: Supplementary file 4 — Supplemental Figures Descriptions [file 41423_2023_1011_MOESM4_ESM.docx]

**Supplementary Figures**

**Supplemental Fig. 1.** **Pharmacological inhibition and genetic deficiency of SCD1 reduces EAE disease severity. (A)** Representative images of CD3 and F4/80 staining of spinal cord tissue obtained from wt (n=6) and *Scd1*^-/-^ (n=5) EAE animals at the peak of the disease (18 dpi). Scale bar: 100 µm. **(B)** Representative images of CD3 and F4/80 staining of spinal cord tissue obtained from vehicle- (n=9) and SCD1 inhibitor-treated (n=10) EAE animals (24 dpi). EAE disease incidence **(C,E,G,I,K)** and onset **(B,D,F,H,J)** of wild-type (wt, n=15) and *Scd1*^-/-^ (n=14) mice (C,D), vehicle- (n=10) and SCD1 inhibitor-treated (SCD1^inh^, 2.5 mg/kg, n=10) mice (E,F), wt (n=19) and *Scd1*^Fl+/+^ *LysM*^Cre+/-^ (n=11) mice (G,H), wt mice that received wt (Wt 🢥 Wt, n=8) or *Scd1*^-/-^ (*Scd1*^-/-^ 🢥 Wt, n=10) encephalitogenic lymphocytes (I,J), wt (n=5) and *Scd1*^-/-^ (n=4) mice that received wt encephalitogenic lymphocytes (K,L). Animals that did not develop EAE were not included in the disease onset analysis. Dpi: days post-immunization. All replicates were biologically independent. All data are represented as mean ± SEM and are pooled from 3 (C) or 2 (E, G, I) independent immunizations. *, P < 0.05; ***, P < 0.001; calculated with two-tailed unpaired student T-test (C-I,K,L), or Mann-Whitney analysis (J).

**Supplemental Fig. 2. *Scd1* deficiency reduces autoimmune-mediated demyelination in a T cell-dependent manner.** **(A-C)** Representative images (A) and quantification (B, C) of MBP and SMI312 staining of spinal cord tissue obtained from wt (n=10) and *Scd1*^-/-^ (n=10) EAE animals (23 dpi). **(A, D, E)** Representative images (A) and quantification (D, E) of MBP and SMI312 staining of spinal cord tissue obtained from wt recipient mice that received lymph node-derived T lymphocytes from immunized wt (Wt 🢥 Wt, n=8) or *Scd1*^-/-^ mice (*Scd1*^-/-^ 🢥 Wt, n=10). All data are represented as mean ± SEM. **, P < 0.01; ***, P < 0.001; calculated with two-tailed unpaired student T-test. **Supplemental Fig. 3. *Scd1* deficiency increases Treg differentiation. (A)** Frequency of CD4^+^, CD8^+^, CD4^-^CD8^-^, CD4^+^IFNγ^+^, CD4^+^IL17^+^, CD4^+^IL4^+^, and CD4^+^FOXP3^+^ in the spleen of wild-type (wt, n=11 animals) and *Scd1*^-/-^ (n=11 animals) EAE animals 10 days after EAE induction. Representative flow cytometric plots are shown. **(B,C)** Representative flow cytometric plots of wt and *Scd1*^-/-^ mouse naïve T cells and human naïve T cells differentiated under Treg polarizing conditions and treated with vehicle or SCD1 inhibitor (SCD1^inh^, CAY10566, 1 µM). For mouse T cell cultures, the frequency of CD4^+^FOXP3^+^ cells was quantified one and two days after Treg induction. For human T cell cultures, frequency of CD25^Hi^FOXP3^+^ cells was quantified four days after induction. **(D)** Wt and *Scd1*^-/-^ mouse naïve T cells were differentiated under Th1, Th2, or Th17 polarizing conditions and treated with vehicle or SCD1 inhibitor (SCD1^inh^, CAY10566, 1 µM). The frequency of CD4^+^IFNγ^+^, CD4^+^IL4^+^, and CD4^+^IL17^+^ cells was quantified two days after Th induction (n=4 samples). All data are represented as mean ± SEM.

**Supplemental Fig. 4. Loss of *Scd1* does not affect the suppressive and proliferative capacity of regulatory T cells. (A,B)** Representative flow cytometric plots of the suppressive capacity of wild-type (wt) and *Scd1^-/-^* mouse Tregs (A), or human Tregs differentiated under the presence of SCD1 inhibitor (SCD1^inh^, CAY10566, 1 µM) or vehicle (B). Increasing amounts of Tregs were cultured with CFSE- or CellTrace Violet (CTV)-labeled CD4^+^CD25^-^ effector T cells (D, n=3 samples; E, n=2 healthy controls). Percentage proliferation was assessed after three (A) or five (B) days. **(C)** Mouse CD4^+^CD25^+^ regulatory T cells were labelled with CFSE, stimulated with anti-CD3ε (2 µg/ml), anti-CD28 (2 µg/ml), recombinant IL-2 (5 U/ml), and analyzed after four days with flow cytometry (n=4 samples). All data are represented as mean ± SEM. ***, P < 0.001; calculated with two-tailed unpaired student T-test.

**Supplemental Fig. 5. Pathway analysis and validation of differentially expressed genes in wt and *Scd1*^-/-^ naïve T cells. (A, B)** Bulk RNA sequencing was performed on wild-type (wt) and *Scd1*^-/-^ naïve T cells. Disorders and canonical pathways associated with *Scd1*^-/-^ T cells identified using Ingenuity Pathway Analysis. **(C)** Validation of mRNA expression of *Prickle1, Acsf2, Pfn2, Bax, Il10, Ifnγ*, *Il4*, *Ptdss1*, *F2rl1*, *Pik3c2a*, *Dapl1*, *Ckap5*, *Rab5a*, *Cd55,* and *Sntb1* in wt and *Scd1*^-/-^ naïve T cells (n=6-12 samples). Data are represented as mean ± SEM. *, P < 0.05; **, P < 0.01, and ***, P < 0.001, calculated with two-tailed unpaired student T-test and corrected for multiple testing with the two-stage step-up method of Benjamin, Krieger and Yekutieli. DEGs: differentially expressed genes. All results are pooled from four independent experiments.

**Supplemental Fig. 6. Transcriptional changes in early and committed *Scd1* deficient Tregs.** **(A, B)** Expression of regulatory T cell (Treg)-associated functional genes *Foxp3*, *Ikzf2*, and *Rgs1* (A), and DHA-signaling genes *Pnpla2*, *Abca1*, *Ptdss1*, and *Pik3c2a* (B) in CD4^+^CD25^+^ Tregs isolated from naive *Scd1^-/-^* mice and wild-type (wt) littermates (n=4 mice/group). **(C-G)** Bulk RNA sequencing was performed on wt and *Scd1^-/-^* early Tregs (n=4 mice/group), which were induced by exposing naïve wt and *Scd1^-/-^* T cells to a Treg differentiation cocktail for a brief period of time (1d). **(C)** Differential gene expression analysis of the RNA sequencing data (log_2_ fold change < -0.5 and >0.5; P value <0.05; complete list in Supplementary Table 3). **(D)** Overlap of significantly up- and downregulated genes in *Scd1^-/-^* naïve T cells compared to early iTregs. **(E)** mRNA expression of *Foxp3*, *Ikzf2*, and *Rgs1* in wt and *Scd1^-/-^* early iTregs. **(F)** Molecular and cellular function categories associated with differentially expressed genes in *Scd1*^-/-^ early iTregs, identified using Ingenuity Pathway Analysis (IPA). **(G)** Canonical pathways associated with *Scd1^-/-^* naïve T cells and early iTregs identified using IPA. Data are represented as mean ± SEM; calculated with two-tailed unpaired student T-test or Mann-Whitney analysis.

**Supplemental Fig. 7. *Scd1*^-/-^ naïve T cells show a decreased intracellular abundance of fatty acid-containing lipids. (A-R)** Liquid chromatography electrospray ionization tandem mass spectrometry (LC-ESI-MS/MS) analysis was used to define the lipidome of wild-type (wt) and *Scd1*^-/-^ naïve T cells (n=2 samples). Log_2_ fold change abundance of fatty acid species within each lipid class is shown (*Scd1*^-/-^ vs. wt). Only detectable lipid species and fatty acyl moieties are shown. **(S)** Flow cytometric analysis of forward scatter (FSC) and sideward scatter (SSC) of wt and *Scd1*^-/-^ naïve T cells. Results are pooled from two (A-R) or 12 (S) independent experiments. Data are represented as mean (A-R) or as mean ± SEM (S); calculated with two-tailed unpaired student T-test.

**Supplemental Fig. 8. ATGL inhibition decreases intracellular non-esterified DHA levels in naïve *Scd1*-deficient T cells. (A)** Liquid chromatography tandem mass spectrometry (LC-MS/MS) analysis was performed to define the abundance of intracellular non-esterified fatty acids and downstream metabolites in *Scd1*^-/-^ naïve T cells treated with vehicle (n=2 samples) or ATGL inhibitor (ATGL^inh^, Atglistatin, 20 µM; n=2 samples) for two days. Log_2_ fold change abundance of all detectable fatty acids and downstream metabolites is shown (Atglistatin-treated vs. vehicle-treated). **(B)** ShRNA-mediated gene silencing of *Atgl* in mouse naïve CD4^+^ T cells.

**Supplemental Fig. 9. PPARγ signaling promotes Treg differentiation in the absence of SCD1. (A)** mRNA expression of PPARγ-response genes *Srebpc1*, *Abca1*, *Cd36*, *Cpt1a* and *Lpl* in wt and *Scd1*^-/-^ naïve T cells (n=3-5 samples). **(B)** Human naïve T cells were differentiated under Treg polarizing conditions and treated with vehicle (n=5 healthy controls) or PPARγ^anta^ (25 μM; n=4 healthy controls). Frequency of CD4^+^FOXP3^+^ cells was quantified four days after Treg induction. Representative flow cytometric plots are shown. **(C-H)** Wt and *Scd1^-/-^* EAE animals were treated daily with a PPARγ-antagonist (GW9662, 2mg/kg, n=5/group) or vehicle, and were sacrificed 28 days after EAE induction. Frequency of CD4^+^, CD8^+^, CD4^-^CD8^-^, CD4^+^IFNγ^+^, CD4^+^IL17^+^, CD4^+^IL4^+^, and CD4^+^FOXP3^+^ cells in the lymph nodes (D). mRNA expression of *Nos2*, *Tnfα*, *Il1β*, *Il6*, *Ccl2*, *Ccl4, and Ccl5* in spinal cord tissue (E). Representative images (E) and quantification (F-H) of CD3, MBP, and SMI312 staining of spinal cord tissue. Data are represented as mean ± SEM. *, P<0.05; **, P < 0.01; ***, P<0.001; calculated with unpaired student T-test (A) and Mann-Whitney U test (E,F).
