# Supplementary figures and images for: Fatty acid desaturation by stearoyl-CoA desaturase-1 controls regulatory T cell differentiation and autoimmunity
Source: Cell Mol Immunol. 2023 Apr 12;20(6):666–79. doi: 10.1038/s41423-023-01011-2 (PMC10229556; doi:10.1038/s41423-023-01011-2)

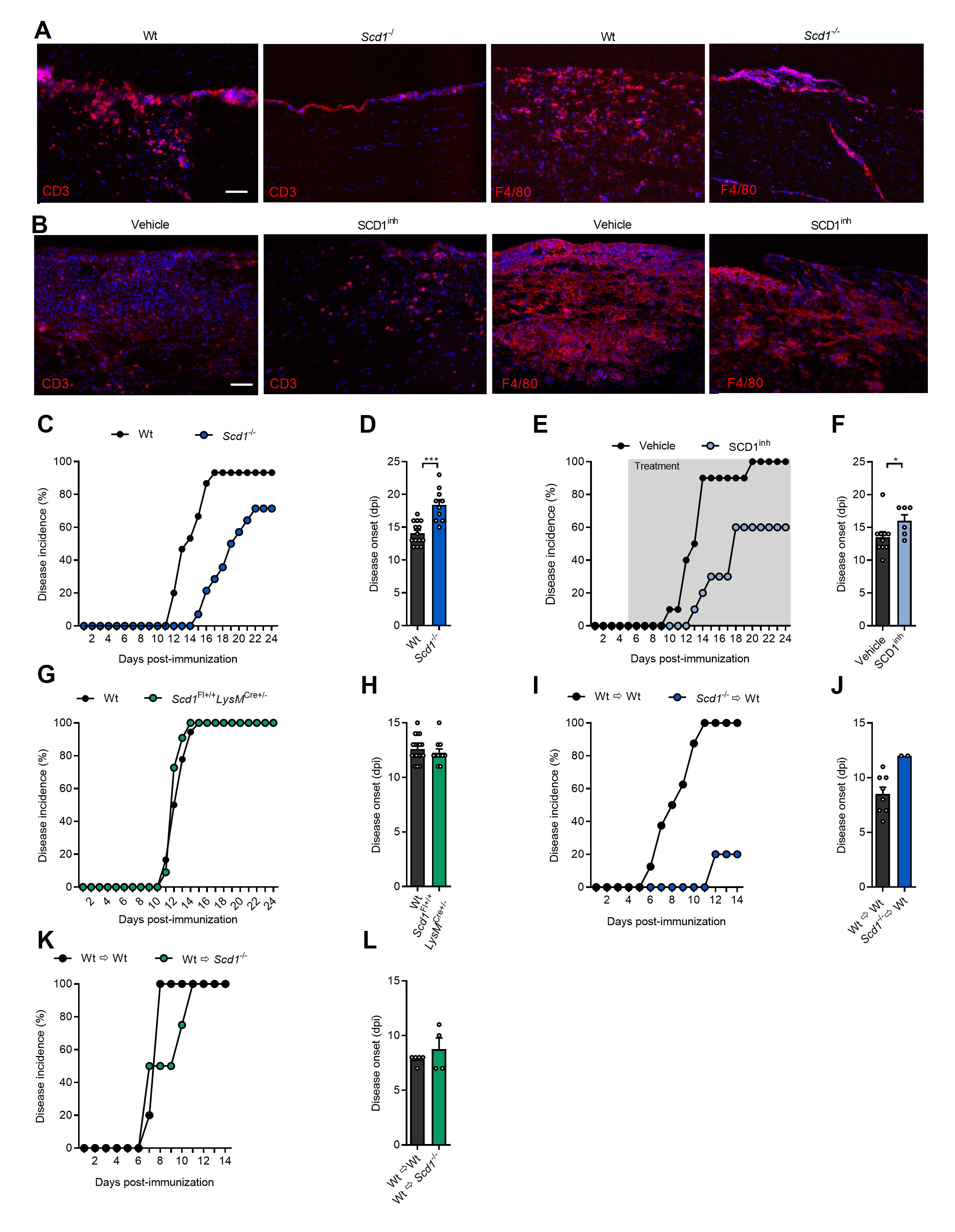

Supplement: Supplementary file 5 — Supplemental Figure 1 [file 41423_2023_1011_MOESM5_ESM.tif]

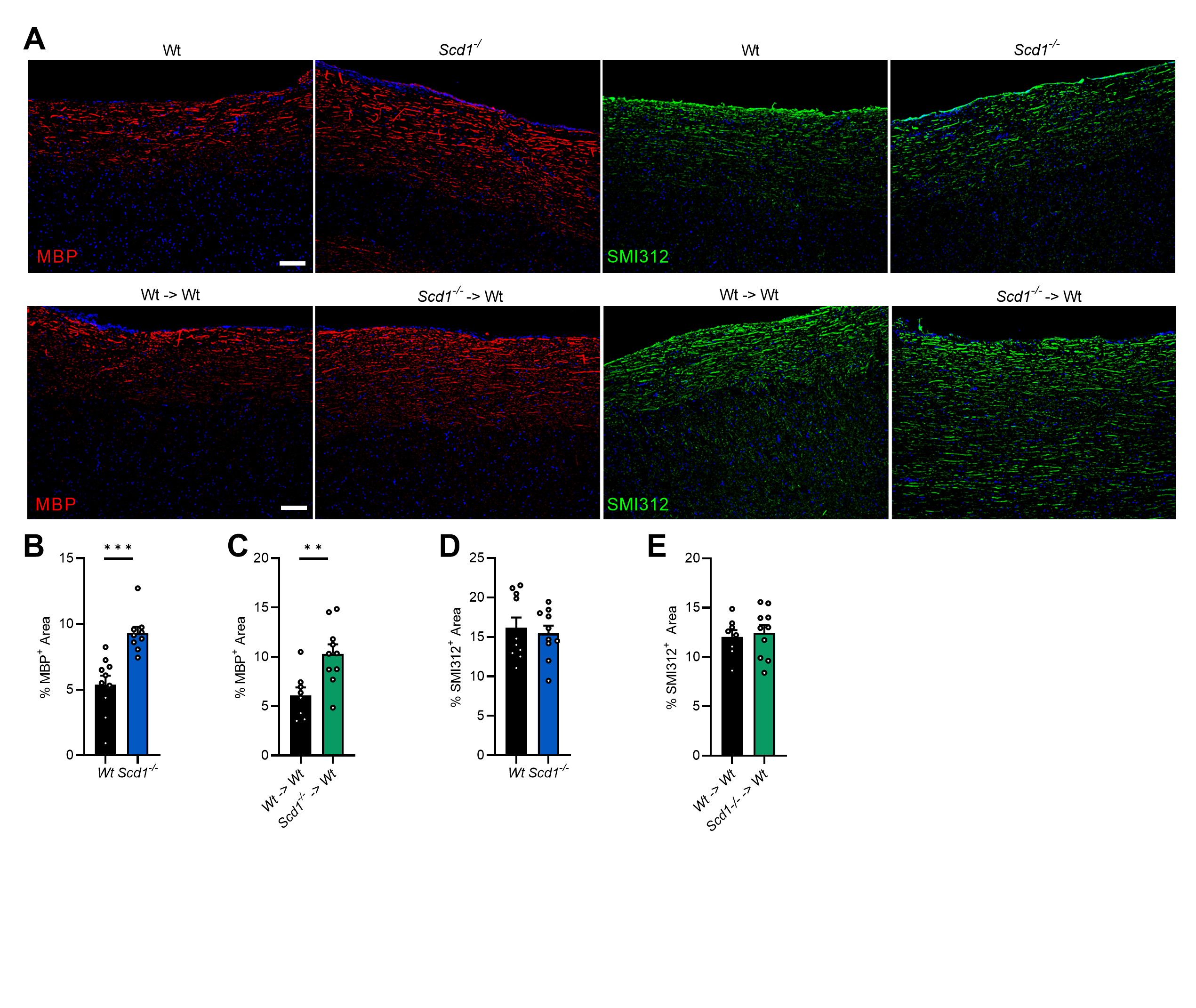

Supplement: Supplementary file 6 — Supplemental Figure 2 [file 41423_2023_1011_MOESM6_ESM.tif]

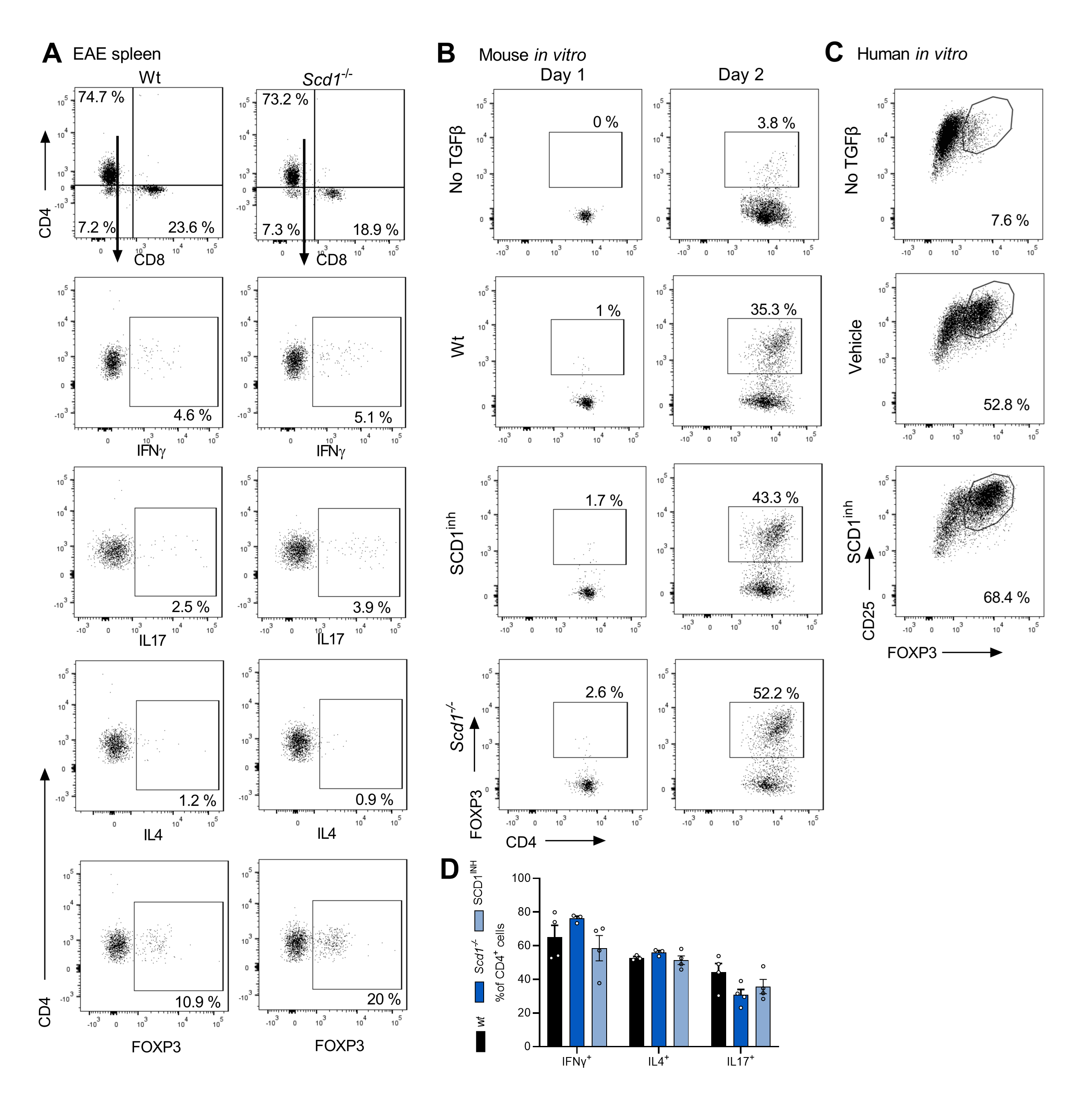

Supplement: Supplementary file 7 — Supplemental Figure 3 [file 41423_2023_1011_MOESM7_ESM.tif]

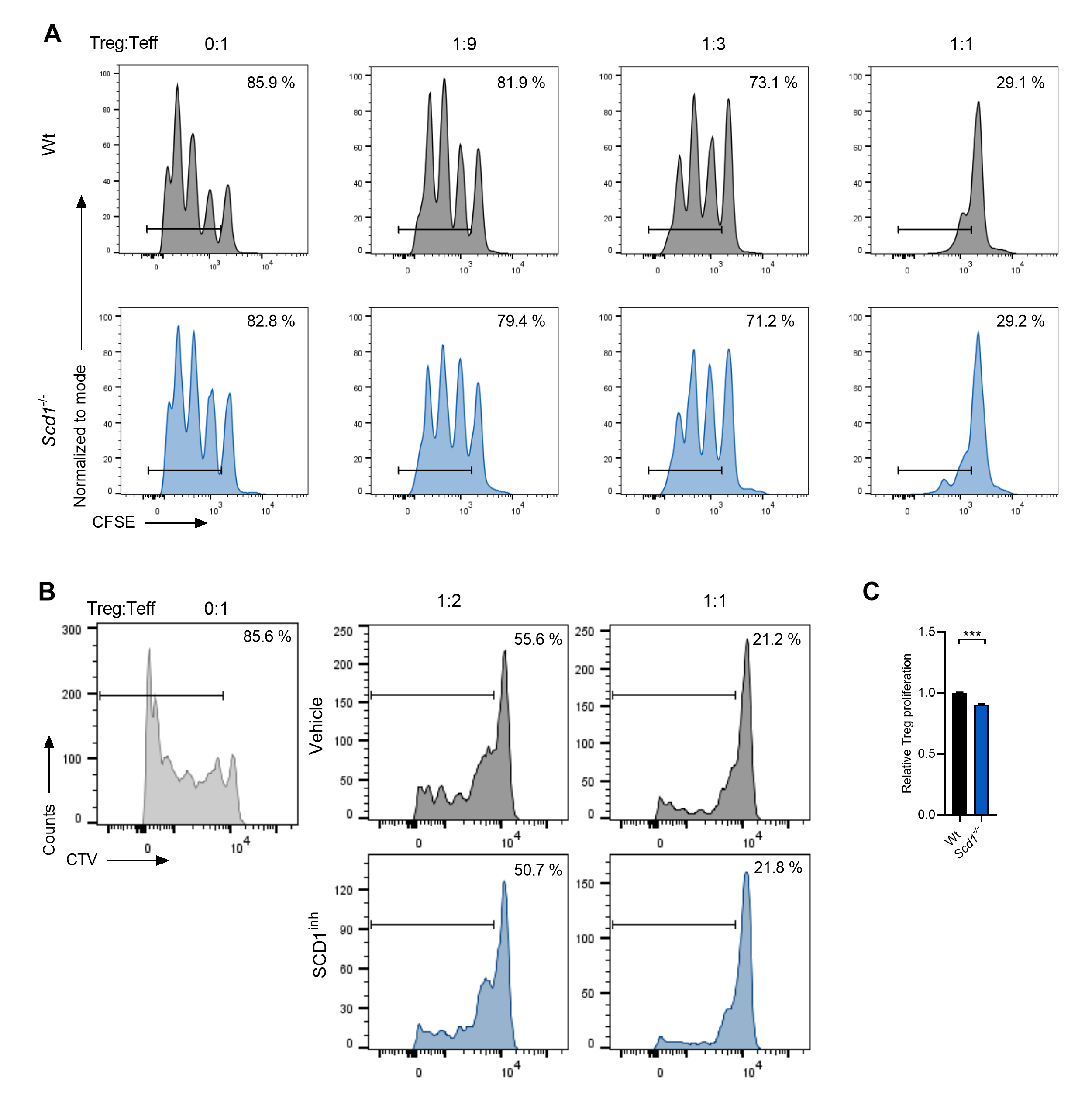

Supplement: Supplementary file 8 — Supplemental Figure 4 [file 41423_2023_1011_MOESM8_ESM.tif]

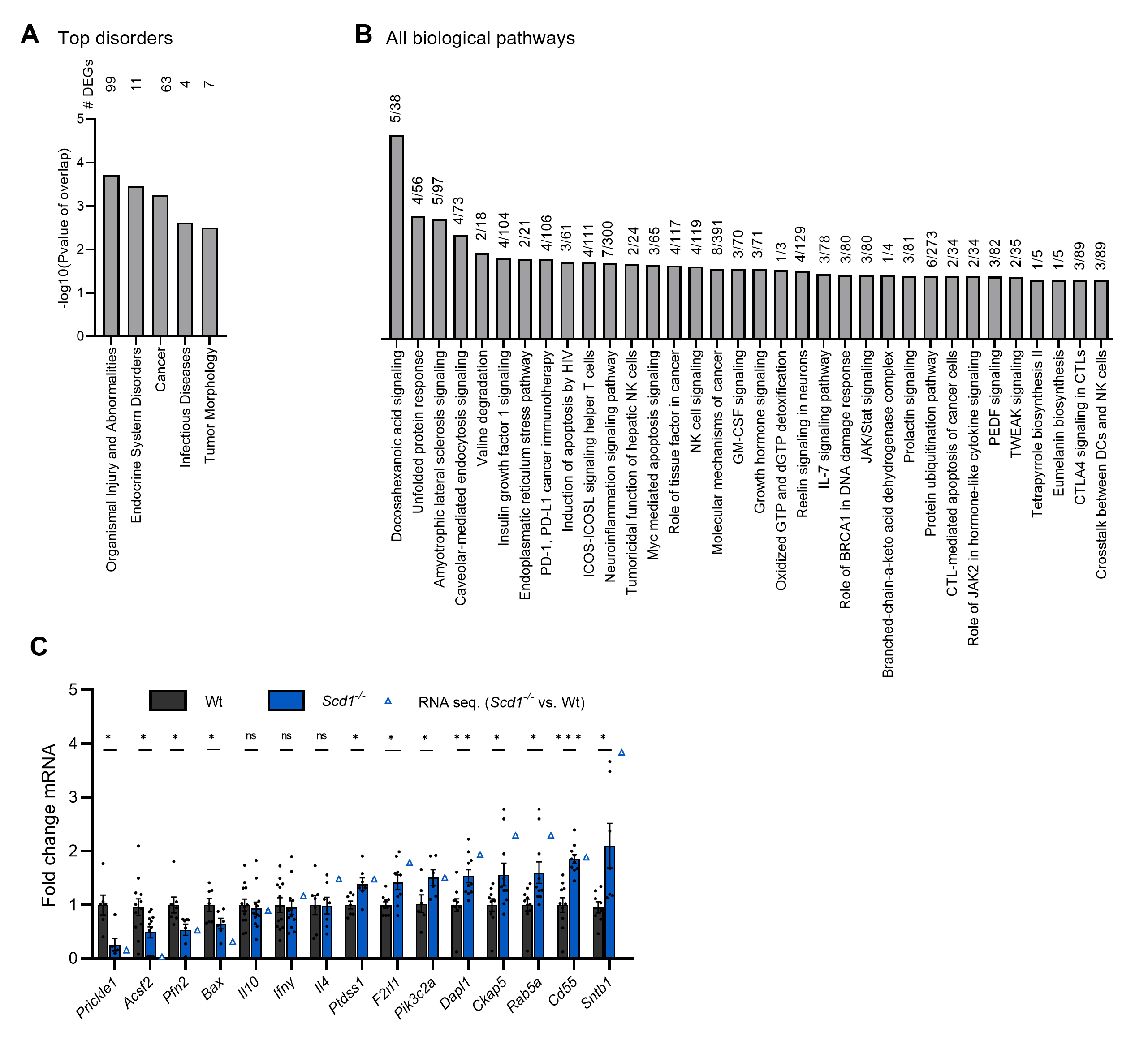

Supplement: Supplementary file 9 — Supplemental Figure 5 [file 41423_2023_1011_MOESM9_ESM.tif]

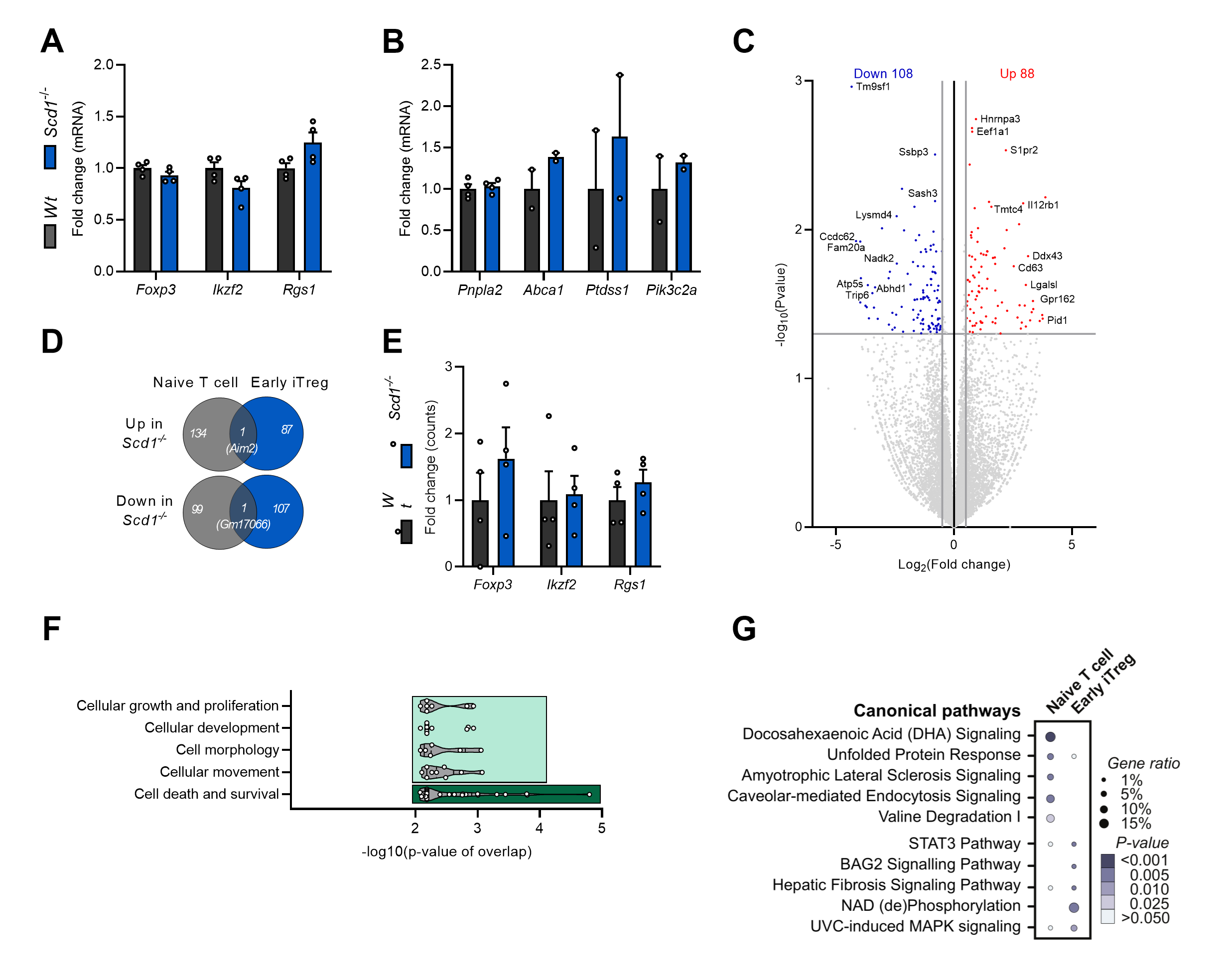

Supplement: Supplementary file 10 — Supplemental Figure 6 [file 41423_2023_1011_MOESM10_ESM.tif]

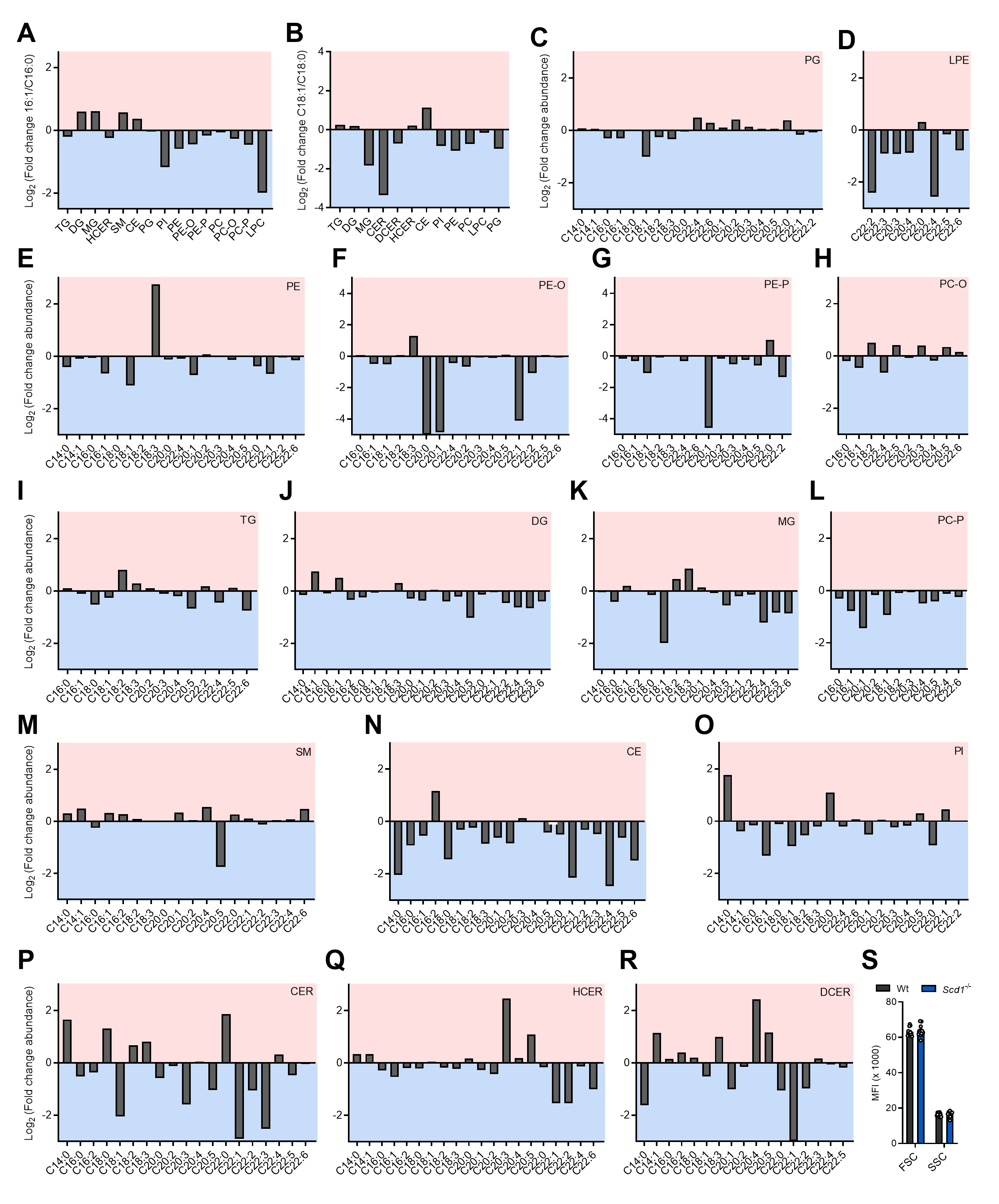

Supplement: Supplementary file 11 — Supplemental Figure 7 [file 41423_2023_1011_MOESM11_ESM.tif]

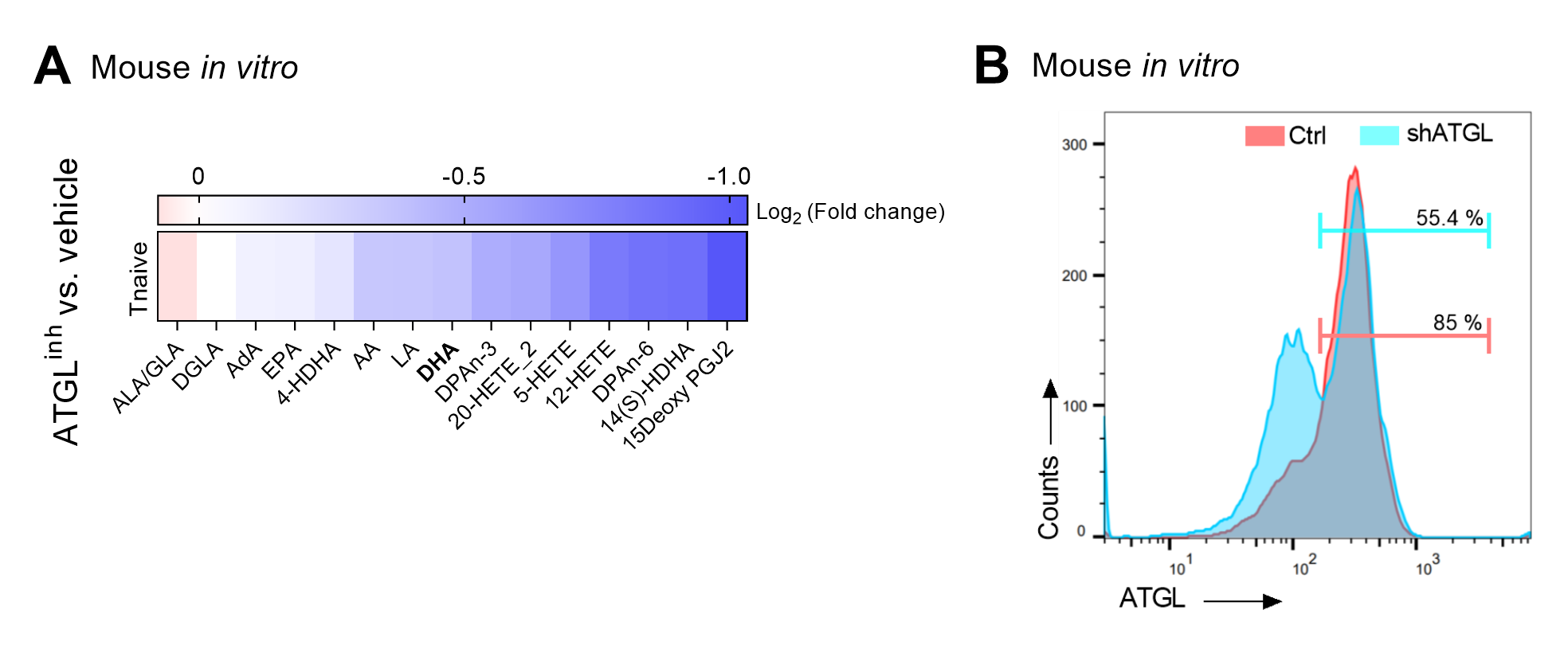

Supplement: Supplementary file 12 — Supplemental Figure 8 [file 41423_2023_1011_MOESM12_ESM.tif]

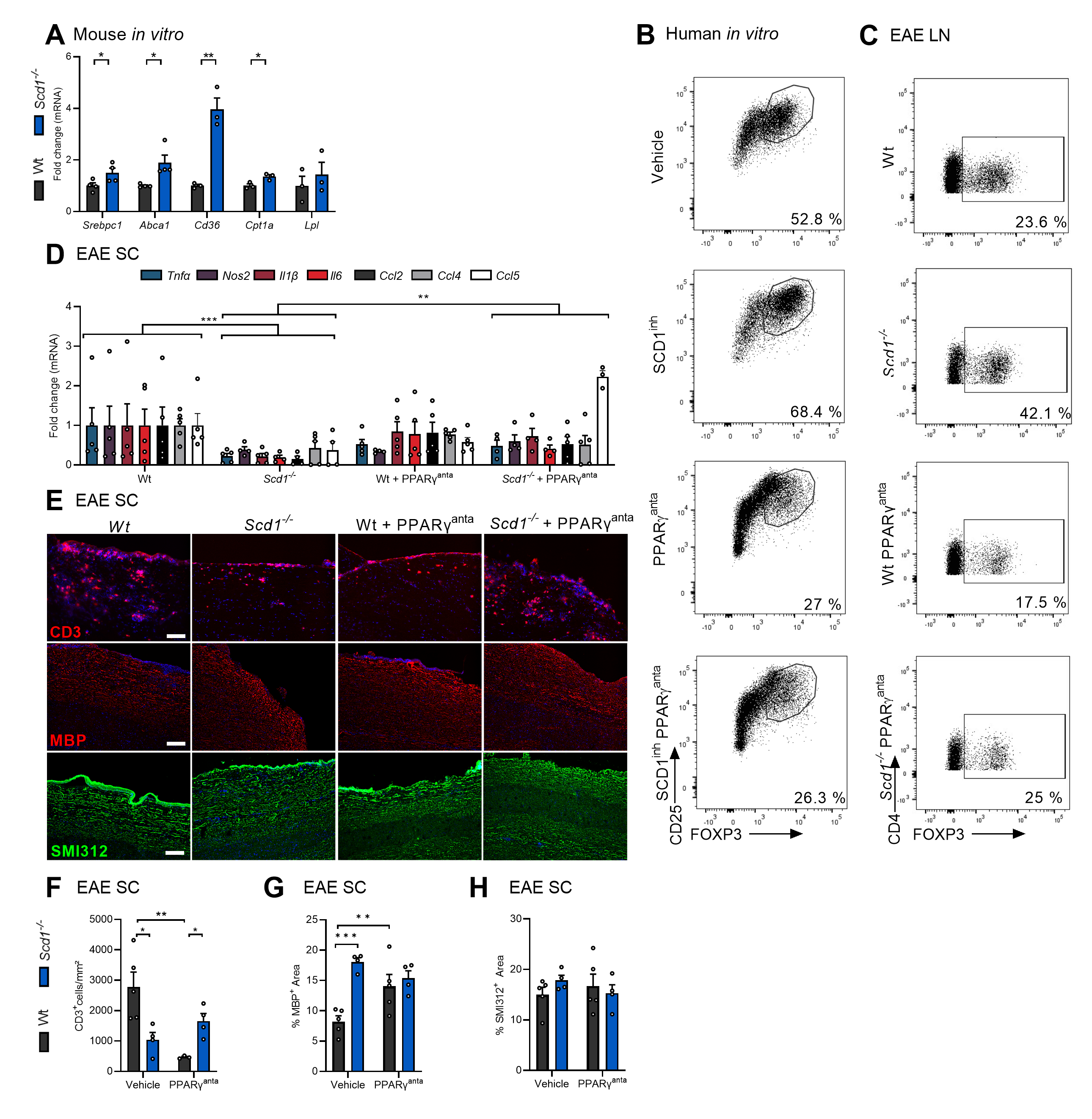

Supplement: Supplementary file 13 — Supplemental Figure 9 [file 41423_2023_1011_MOESM13_ESM.tif]
